# Accumulation of and Response to Auxins in Roots and Nodules of the Actinorhizal Plant *Datisca glomerata* Compared to the Model Legume *Medicago truncatula*

**DOI:** 10.3389/fpls.2019.01085

**Published:** 2019-09-24

**Authors:** Irina V. Demina, Pooja Jha Maity, Anurupa Nagchowdhury, Jason L. P. Ng, Eric van der Graaff, Kirill N. Demchenko, Thomas Roitsch, Ulrike Mathesius, Katharina Pawlowski

**Affiliations:** ^1^Department of Ecology, Environment and Plant Sciences, Stockholm University, Stockholm, Sweden; ^2^Division of Plant Science, Research School of Biology, Australian National University, Canberra, ACT, Australia; ^3^Department of Plant Physiology, Karl-Franzens-Universität Graz, Graz, Austria; ^4^Laboratory of Cellular and Molecular Mechanisms of Plant Development, Komarov Botanical Institute, Russian Academy of Sciences, Saint-Petersburg, Russia; ^5^Laboratory of Molecular and Cellular Biology, All-Russia Research Institute for Agricultural Microbiology, Saint-Petersburg, Russia

**Keywords:** IAA, PAA, cytokinins, *DR5:GUS*, hairy roots, root branching, nodules

## Abstract

Actinorhizal nodules are structurally different from legume nodules and show a greater similarity to lateral roots. Because of the important role of auxins in lateral root and nodule formation, auxin profiles were examined in roots and nodules of the actinorhizal species *Datisca glomerata* and the model legume *Medicago truncatula*. The auxin response in roots and nodules of both species was analyzed in transgenic root systems expressing a beta-glucuronidase gene under control of the synthetic auxin-responsive promoter *DR5*. The effects of two different auxin on root development were compared for both species. The auxin present in nodules at the highest levels was phenylacetic acid (PAA). No differences were found between the concentrations of active auxins of roots *vs.* nodules, while levels of the auxin conjugate indole-3-acetic acid-alanine were increased in nodules compared to roots of both species. Because auxins typically act in concert with cytokinins, cytokinins were also quantified. Concentrations of *cis*-zeatin and some glycosylated cytokinins were dramatically increased in nodules compared to roots of *D. glomerata*, but not of *M. truncatula*. The ratio of active auxins to cytokinins remained similar in nodules compared to roots in both species. The auxin response, as shown by the activation of the *DR5* promoter, seemed significantly reduced in nodules compared to roots of both species, suggesting the accumulation of auxins in cell types that do not express the signal transduction pathway leading to *DR5* activation. Effects on root development were analyzed for the synthetic auxin naphthaleneacetic acid (NAA) and PAA, the dominant auxin in nodules. Both auxins had similar effects, except that the sensitivity of roots to PAA was lower than to NAA. However, while the effects of both auxins on primary root growth were similar for both species, effects on root branching were different: both auxins had the classical positive effect on root branching in *M. truncatula*, but a negative effect in *D. glomerata*. Such a negative effect of exogenous auxin on root branching has previously been found for a cucurbit that forms lateral root primordia in the meristem of the parental root; however, root branching in *D. glomerata* does not follow that pattern.

## Introduction

Plant productivity depends on the successful acquisition of water and nutrients from the soil. Therefore, when exposed to nitrogen limitation, certain groups of plants enter a symbiosis with nitrogen-fixing soil bacteria. In the symbiosis, specialized lateral root organs called the root nodules are formed and serve for the stable internal accommodation of the bacterial microsymbionts. There are two main types of root nodule symbioses: rhizobia enter symbioses with legumes and with *Parasponia* sp. (Cannabaceae, Rosales), and soil actinobacteria of the genus *Frankia* enter so-called actinorhizal symbioses with a diverse group of dicotyledonous plants from eight families from three orders (Datiscaceae and Coriariaceae from the Cucurbitales; Betulaceae, Casuarinaceae, and Myricaceae from the Fagales; and Elaeagnaceae, Rhamnaceae, and Rosaceae from the Rosales).

Both nodules and lateral roots are formed postembryonically from preexisting roots. The initiation of both involves reactivation of the cell cycle and subsequent redifferentiation (Demchenko and Demchenko, 2001; Gage, 2004; Voroshilova et al., 2009). Legume and actinorhizal nodules differ in anatomy and ontogeny. Legume nodules are typically stem-like organs in that they contain a peripheral vascular system, while rhizobia-infected cells are located in the inner tissue. In contrast, actinorhizal nodules and rhizobia-induced nodules of the non-legume *Parasponia* are coralloid organs composed of multiple lobes, each of which represents a modified lateral root with a central vascular system and *Frankia*-infected cells in the nodule cortex, surrounded by a periderm. Legume nodule primordia are initiated in the cortex and pericycle, while actinorhizal nodule primordia are initiated in the pericycle like lateral root primordia (Pawlowski and Bisseling, 1996). Many legume nodules, e.g., from *Medicago truncatula*, *Cicer arietinum*, and *Vicia faba*, and all actinorhizal nodule lobes are of the indeterminate type, i.e., they have an apical meristem whose function leads to the formation of a developmental gradient of infected cells in the nodule inner tissue (legume nodules) or nodule cortex (actinorhizal nodules; Pawlowski and Bisseling, 1996).

Nodulation is initiated by bacterial signals, which have been extensively characterized for rhizobia, but not for frankiae. With a few exceptions, rhizobial nodulation signals represent lipochitooligosaccharides termed Nod factors. Upon perception of Nod factors by cells of the root epidermis, signaling *via* the symbiosis receptor kinase SymRK leads to calcium spiking in the nucleus and results in the activation of cytokinin signaling (Oldroyd et al., 2011). Cytokinin signaling leads to increased auxin signaling in the root pericycle and cortex, which is likely necessary for nodule initiation in legumes (Suzaki et al., 2012; Ng et al., 2015). Whether this is the case for actinorhizal nodules is still uncertain. However, auxin is a very likely actor during actinorhizal nodule development because numerous studies have shown auxin’s essential role in lateral root formation, where it also interacts with cytokinin (e.g., Bishopp et al., 2011).

To monitor the auxin response during root and nodule development, the expression patterns of a fusion of an auxin-inducible promoter (i.e., *GH3* or *DR5*; Ulmasov et al., 1995; Ulmasov et al., 1997) and the reporter gene β-glucuronidase (*GUS*; Jefferson et al., 1987) were studied during legume nodule development. During the early stages of nodulation, auxin signaling was shown to be activated in root hairs and to be necessary for infection in *M. truncatula* (Breakspear et al., 2014). Similarly, in the determinate nodule-forming model legume *Lotus japonicus*, it could be shown that Nod factor signaling also induces auxin biosynthesis and accumulation in infected root hairs and, in turn, auxin biosynthesis promotes infection events and restricts cytokinin signaling (Nadzieja et al., 2018). In *Trifolium repens* and *M. truncatula* roots expressing a phytohormone-inducible promoter-*GUS* fusion, rhizobia and purified Nod factors inhibited the auxin response at and below the site of infection preceding nodule primordium formation (Mathesius et al., 1998; Huo et al., 2006; van Noorden et al., 2007). The decrease in promoter activity was followed by its increase in the cortex at the site of nodule initiation and then by its consequent disappearance from the central tissue of the differentiating nodule. Altogether, the requirement for auxin during indeterminate legume nodulation changes over time, requiring high auxin responses at the first stages of cell divisions but subsequently lower auxin responses during nodule differentiation, similar to the auxin requirements during the induction of lateral roots (Mathesius et al., 1998). In mature indeterminate nodules, e.g., of *T. repens* and *M. truncatula*, the expression of auxin responsive reporters was confined to vascular tissues and the apical meristem (Mathesius et al., 1998; Guan et al., 2013; Breakspear et al., 2014; Franssen et al., 2015), while in determinate nodules of *L. japonicus* and *Glycine max*, which do not maintain an apical meristem, auxin responses are found mainly in the vascular tissue (Takanashi et al., 2011; Suzaki et al., 2012; Turner et al., 2013; Fisher et al., 2018). Similar to *GH3*, *MtLAX*, which encodes an auxin influx carrier, was expressed in the nodule vasculature in *M. truncatula* (de Billy et al., 2001). These expression patterns support a role of auxin in vascular differentiation and nodule meristem maintenance (Mathesius et al., 1998; Fisher et al., 2018). A combined reporter for auxin and cytokinin responses in soybean has also shown that the ratios of auxin to cytokinin change in different nodule zones, with high auxin to cytokinin ratios in nodule vascular tissue but low auxin to cytokinin ratios in the infection zone of nodules (Fisher et al., 2018).

In contrast to the above-mentioned studies in legumes, auxin has been studied to a lesser extent in actinorhizal species. Application of a synthetic auxin influx inhibitor delayed nodule formation in *Casuarina glauca* (Péret et al., 2007) and auxin accumulated in infected cortical cells of nodules of the intracellularly infected species *C. glauca* (Perrine-Walker et al., 2010). However, in nodules of the intercellularly infected species *Discaria trinervis*, auxin seemed to accumulate in non-infected cortical cells (Imanishi et al., 2014).

Less is known about auxins in mature actinorhizal nodules. The indole-3-acetic acid (IAA) content of nodules and roots was quantified for *Alnus glutinosa* and *C. glauca* (Wheeler et al., 1979; Perrine-Walker et al., 2010). In *C. glauca*, immunolocalization of IAA showed that it accumulated in nodule cells containing frankiae (Perrine-Walker et al., 2010). Moreover, the activity of the promoter of the auxin influx gene *CgAUX1* was confined to *Frankia*-infected cells throughout the course of nodule development (Péret et al., 2007). In contrast, no promoter activity of *CgAUX1* was detected in root cells colonized by the arbuscular mycorrhizal fungus *Glomus intraradices* (Péret et al., 2008), indicating that the activity of the *CgAUX1* promoter during plant cell infection was a specific response to frankiae (Péret et al., 2008).

The action of auxin is typically coupled to the action of cytokinin, another phytohormone essential for nodulation. The cytokinin receptor kinase responsible for the activation of the cytokinin response to Nod factors was termed MtCRE1 (cytokinin response 1) in *M. truncatula* (Gonzalez-Rizzo et al., 2006) and LHK1 in *L. japonicus* (Murray et al., 2007; Tirichine et al., 2007). A loss-of-function mutation of *MtCRE1* led to reduced nodulation, while a constitutive mutation of *CRE1* or *LHK1* caused spontaneous nodule formation in the absence of rhizobia (Tirichine et al., 2007; Ovchinnikova et al., 2011). Furthermore, cytokinin applied exogenously or constitutively secreted by Nod^−^ rhizobial strains was sufficient to activate cortical cell divisions and initiate some of the morphological and molecular events typical for nodulation (Cooper and Long, 1994; Mathesius et al., 2000). This response is only found in nodulating legumes but not in non-nodulating species nor in actinorhizal species (Gauthier-Coles et al., 2019). Cytokinin synthesis genes are activated during nodulation in various legumes (see, e.g., Mortier et al., 2014; Dolgikh et al., 2017; Reid et al., 2017), and increased cytokinin concentrations were measured in the root zone susceptible to rhizobia within 3 h after application of Nod factors (van Zeijl et al., 2015). Altogether, these data demonstrate that, in legumes, cytokinin signaling is necessary and sufficient for the activation of cortical cell divisions that lead to nodule organogenesis. A direct result of cytokinin signaling is an MtCRE1-dependent change in polar auxin transport that is mediated by flavonoids (Plet et al., 2011; Ng et al., 2015). This is consistent with the fact that external application of synthetic auxin transport inhibitors to plant roots induced the formation of nodules in the absence of rhizobia (Allen et al., 1953; Hirsch et al., 1989; Rightmyer and Long, 2011), although this response is only found in legumes forming indeterminate nodules (Ng and Mathesius, 2018). However, the roles and distribution of cytokinins in mature legume nodules have not been studied in detail.

In actinorhizal symbiosis, the role of cytokinins in nodule formation has not been studied. Homologues of the *MtCRE1* gene were shown to be transcribed in roots and nodules of *C. glauca* (Fagales) and nodules of *Datisca glomerata* (Cucurbitales; Hocher et al., 2011; Demina et al., 2013). Cytokinin quantifications have been performed for nodules and roots of three species: *A. glutinosa* (Betulaceae, Fagales), *Myrica gale* (Myricaceae, Fagales), and *Purshia tridentata* (Rosaceae, Rosales; Wheeler et al., 1979).

Actinorhizal symbioses are very diverse, and most of the available data on phytohormones and their role in nodule development are on *A. glutinosa* and *C. glauca* (Fagales). This study was aimed at broadening our view on auxin in nodulation and root development by examining another actinorhizal species, *D. glomerata* (Datiscaceae, Cucurbitales), the structure of whose nodules differs from that of actinorhizal Fagales and Rosales (Pawlowski and Demchenko, 2012). For comparisons, we used the indeterminate-nodule forming model legume *M. truncatula*. First, we performed quantifications of auxins in non-inoculated roots and in nodules of *M. truncatula* and *D. glomerata*. Cytokinins were also quantified to gain insight into the relative changes of auxins and cytokinins in actinorhizal and legume nodules. Second, to gain insights into the distribution of auxin responses in nodulation, we studied *DR5* promoter activity in nodulated and non-nodulated transgenic hairy roots of *M. truncatula* and *D. glomerata*. Lastly, we investigated the auxin response of roots in these two plant species by exogenous application of the synthetic auxin 1-naphthaleneacetic acid (NAA) and the natural auxin phenylacetic acid (PAA) to seedlings grown in axenic culture. PAA was chosen because it had turned out to be the dominant auxin of roots and nodules of *M. truncatula* and *D. glomerata*. While the effect of auxin on nodulation can easily be studied in legumes under *in vitro* conditions, this was not possible in *D. glomerata* due to the fact that plants can only be grown in soil and nodules form slowly and asynchronously; inoculation from crushed nodules, due to the unculturable nature of the symbiont, prevent reliable quantification of the inoculum. Therefore, auxin response was measured through its effect on lateral root formation and root elongation, similar to auxin response assays typically carried out in in non-legumes (Woodward and Bartel, 2005).

## Materials and Methods

### Plant Material

*Medicago truncatula* Gaertn. cv. Jemalong genotype A17 was used. Seeds were scarified with sandpaper and germinated on germination soil (S-jord, Hasselfors Garden AB, Sweden). Plants were grown in the greenhouse under a 15-h photoperiod and day/night temperatures of 22°C/19°C, with a light intensity of 70–300 μE m^−2^ s^−1^. Plants were watered once per week with water and once per week with Fåhraeus medium (Fåhraeus, 1957). For nodulation, plants were inoculated with *Sinorhizobium meliloti* 1021 at OD_600_ = 0.1. For auxin quantifications, *M. truncatula* seeds were scarified with sandpaper, surface sterilized in 6% (w/v) sodium hypochlorite for 30 min, rinsed five times in sterile water, and grown on 15-cm diameter nutrient agar containing nitrogen-free Fåhraeus medium [0.9 mM CaCl_2_, 0.5 mM MgSO_4_, 0.7 mM KH_2_PO_4_, 0.8 mM Na_2_HPO_4_, 20 μM iron (II) citrate, supplemented with 0.1 mg/ml of each of the following microelements: MnCl_2_, CuSO_4_, ZnCl_2_, HBO_3_, and Na_2_MnO_4_]. Plants were grown in a growth room at 22°C with a 16-h day length and ∼150 μE m^−2^ s^−1^ light intensity, and nodules were harvested at 6 weeks postinoculation with *S. meliloti* strain 1021.

*Datisca glomerata* (C. Presl.) Baill. seeds were collected from greenhouse plants originating from plants growing in Vaca Hills (California, USA). Seeds were germinated on sand wetted with water; plantlets were transferred to pot soil (S-jord, Hasselfors Garden AB, Hasselfors, Sweden). Plants were grown in a growth chamber under a 15-h photoperiod and day/night temperatures of 25°C/19°C, with a light intensity of 60–100 μE m^−2^ s^−1^ and 65% relative humidity. When the plants had reached a height of *∼*20 cm, they were transferred to bigger pots containing soil (S-jord, Hasselfors Garden AB) and crushed nodules harvested from older *D. glomerata* plants. Plants were watered twice per week, once with water and once with 1/4 strength Hoagland’s medium (Hoagland and Arnon, 1938) without a nitrogen source prepared as follows: 2 mM K_2_SO_4_, 2 mM KH_2_PO_4_, 2.5 mM CaSO_4_, 2 mM MgSO_4_, microelements (2.86 mg/l H_3_BO_3_, 1.81 mg/l MnCl_2_ 4 H_2_O, 0.22 mg/l ZnSO_4_ 7 H_2_O, 0.08 mg/l CuSO_4_ 5 H_2_O, 0.025 mg/l Na_2_MnO_4_ 2 H_2_O, 0.025 mg/l CoCl_2_ 6 H_2_O), 100 ml/lchelated iron stock (20 mM FeSO_4_ and 20 mM Na_2_EDTA), pH 5.8. Nodules were harvested 6–8 weeks after infection.

*Coriaria myrtifolia* L. seeds collected in Jijel (Algeria) were kindly provided by Amir Ktari (University of Carthage, Tunis, Tunisia). They were vernalized on wetted sand at 7°C for 1 week before transfer to the greenhouse to germination soil. Growth conditions were the same as for *D. glomerata*.

*Casuarina glauca* Sieber ex Spreng. seeds were obtained from New Zealand Tree Seeds (nzseeds.co.nz), germinated and grown on germination soil for 3 months before being transferred to pots containing perlite/vermiculite (50/50, v/v) wetted with 1/4 strength Hoagland’s medium containing 10 mM KNO_3_ (Hoagland and Arnon, 1938). Growth conditions were as described for *D. glomerata*. Roots were harvested when the plants were ∼20 cm high.

*Glycine max* (L.) Merr. cv. “Bragg” (soybean) seeds were obtained from Brett Ferguson (University of Queensland, Australia) and inoculated with *Bradyrhizobium japonicum* USDA110 (OD_600_ = 0.1).

*Lotus japonicus* L. cv. Gifu seeds were purchased from the University of Miyazaki (Japan) and inoculated with *Mesorhizobium loti* MAFF303099 at OD_600_ = 0.1. Soybean plants were grown in 2 l pots in sand, while *L. japonicus* was grown in Petri dishes (15 cm diameter) on 1/4 strength B&D medium (Broughton and Dilworth, 1971). Both species took 2 days to germinate and were inoculated 1 week postgermination. Nodules were collected 3 weeks postinoculation in both species. Temperature in the greenhouse was maintained at 25°C during the day and 20°C at night, with a natural day–light cycle.

*Cucumis sativus* L. (cucumber) seeds were purchased from “Seeds2freedom” (Australia) and grown in sand in rectangular 500-ml pots for 3 weeks prior to tissue harvest.

*Begonia bowerae* Ziesenh. cv. Cleopatra was a gift from the glasshouse facility manager at the Research School of Biology (Australian National University, Canberra, Australia). The adult begonia plant was grown in a 2-L pot in a generic soil mixture. Temperature in the greenhouse was maintained at 25°C during the day and 20°C at night, with a natural day light cycle.

*Cicer arietinum* L. (chickpea) seeds (kindly provided by Dr Angela Pattison, University of Sydney) were sterilized in 0.5% bleach for 30 min, coated with P-Pickel T fungicide (Nufarm Australia), and germinated on 1% agar plates over 2 days. Germinated *C. arietinum* seeds were transferred into autoclaved sand in individual 300-ml pots. After 1 week, each seedling was inoculated with 1 ml of *Mesorhizobium ciceri* strain CC1192 (OD_600_ = 0.1). Nodules were harvested 6 weeks postinoculation, snap frozen, and stored until processing.

### Bacterial Material

All rhizobial strains were maintained on TY medium. *Escherichia coli* strain TOP10 (Invitrogen, Stockholm, Sweden) was used for plasmid propagation and manipulation. Strains TOP10 and GJ23 helper (Van Haute et al., 1983) were used for triparental mating. *E. coli* bacteria were grown in Luria–Bertani medium (LB; Miller, 1972) at 37°C. *Agrobacterium rhizogenes* strains R1000 (Moore et al., 1979) and Arqua1 (Quandt et al., 1993) were used for hairy root transformation of *M. truncatula*, and LBA1334 (Offringa et al., 1986) and AR1193 (Stougaard et al., 1987) for hairy root transformation of *D. glomerata*. Agrobacteria were grown in YEB medium (Van Larebeke et al., 1977) at 28°C. *M. truncatula* plants were inoculated with the strain *S. meliloti* Sm1021 (Meade and Signer, 1977). *D. glomerata* plants were inoculated with the strain *Candidatus* Frankia datiscae Dg1, originating from nodules of *Coriaria nepalensis* from Pakistan (Mirza et al., 1994; Persson et al., 2011; Persson et al., 2015). Crushed *D. glomerata* nodules were used for the inoculation of root systems.

### Root Auxin Response Assays

*M. truncatula* seeds were scarified with sandpaper, surface sterilized in 6% (w/v) sodium hypochlorite for 30 min, rinsed five times in sterile water, placed on 1/2 Hoagland’s 1% agar plates (Hoagland and Arnon, 1938), and incubated at 4°C overnight. Hoagland’s medium with a nitrogen source was prepared according to the following protocol: 1 mM KH_2_PO_4_, 2 mM MgSO_4_, 5 mM Ca(NO_3_)_2_, 5 mM KNO_3_, microelements (as above), 100 ml/l chelated iron stock (as above), pH 5.8. *D. glomerata* seeds were surface sterilized by gentle shaking in 70% (v/v) EtOH/0.05% (w/v) sodium dodecyl sulfate (SDS) for 5 min, followed by 20 min incubation in 2.5% (w/v) sodium hypochlorite containing 0.1% (w/v) SDS. Seeds were rinsed five times in sterile water, placed in an Eppendorf tube covered with sterile water, and incubated at 4°C for 1 week. Afterwards, seeds were placed on 1/2 Hoagland’s medium plates with 1% agar. When seedlings had grown to a length of ∼2 cm, they were transferred to 1/2 Hoagland’s 1% agar plates containing various concentrations of PAA or NAA for 28 days. For each auxin concentration, 9−12 plants were analyzed. Plants were grown in a growth room at 22°C with a 16-h day length and ∼150 μEm^−2^ s^−1^ light intensity. At the time of transfer to the hormone-containing plates, the root tip was marked on the plate. Root length was measured relative to the position of the root tip. Emerged and unemerged lateral roots were counted along the whole length of the root, including the 2 cm of the root that had formed at the time of transfer to hormone plates. For the counting of unemerged lateral root primordia under a light microscope, roots were bleached in 6% (w/v) sodium hypochlorite for 3–10 min, followed with rinsing in water and staining in 0.05% (w/v) methylene blue for 5 min. Roots were rinsed in water again before mounting on slides in water.

### Molecular Cloning

Destination vectors used were the pKGW-RR-MGW binary vector (Op den Camp et al., 2011) and an integration vector derived from pIV10 (Radutoiu et al., 2005). An *A. rhizogenes* strain R1000 carrying *DR5*-KGW-RR-MGW was available (Ilina et al., 2012). The vector pKGW-RR-MGW showed no background expression in *M. truncatula* hairy roots (E. Limpens, personal communication). However, when tested in *D. glomerata* in the present study, pKGW-GGRR showed extensive background *GUS* expression in hairy roots (data not shown). Therefore, the pIV10 vector (Radutoiu et al., 2005) was chosen for the preparation of a new *DR5:GUS* fusion construct. The promoter *DR5* was PCR amplified from pBI101.3 (Jefferson et al., 1987) with the primers 5’-ACGGATCCGGTATCG-CAGCCCCCTTTTGTCTC-3’ and 5’-ACCTCGAGGGT-CTTG-CGGGGCTGCA-GG-3’. The *Bam*HI/*Xho*I-digested *proDR5* promoter fragment was subcloned in the *Bam*HI/*Xho*I-digested entry vector pUC18-entry8 (Hornung et al., 2005). Recombination was confirmed with PCR with the forward gene-specific primer 5’-ACGGATCCGGT-ATCGCAGCCCCCTTTTGTCTC-3’ and the *M13* reverse primer 5’-CAGGAAA-CAGCTGAC-3’. Then, *DR5* was transferred from pUC18-entry8 into the integration vector pIV10 upstream of the *GUS* reporter gene ORF to yield the *DR5:GUS* fusion, using the Gateway cloning technology (Clontech, Mountain View, CA, USA). Recombination was confirmed with PCR with the forward gene-specific primer and the *EcGUS* reverse primer 5’-CCGGCTTTCTTGTAACGC-3’. The positive recombinants were sequenced (Eurofins, Ebersberg, Germany) using the *EcGUS* reverse primer. The pIV10 vector with the *DR5:GUS* construct was integrated into *A. rhizogenes* AR1193 T_L_-DNA segment *via* triparental mating (Radutoiu et al., 2005). Selection of integration events was carried out on YEB agar medium containing 100 µg/ml ampicillin, 100 µg/ml spectinomycin, and 100 µg/ml rifampicin. Selected transformants were confirmed by colony PCR or liquid culture PCR with the forward gene-specific primer and the *EcGUS* reverse primer.

### *Agrobacterium rhizogenes*-Mediated Transformation of *M. truncatula* and *D. glomerata*

For *DR5:GUS* expression studies, axenically grown *M. truncatula* plants were transformed with *A. rhizogenes* R1000 as described by Boisson-Dernier et al. (2001). One week prior to inoculation with *S. meliloti*, plants were transferred to slanted 1% agar containing nitrogen-free Fåhraeus medium. Nodules were harvested at different stages of development.

*D. glomerata* transformation was performed using a protocol based on Markmann et al. (2008) with several modifications. Surface-sterilized *D. glomerata* seeds were germinated on Petri dishes with 1/4 strength Hoagland’s medium pH 5.8 containing 5 mM KNO_3_, 10 g/l sucrose, and 0.8% micro agar (Duchefa, Haarlem, The Netherlands) and incubated in the dark for 7 days at 25°C. Afterwards, the plates were placed horizontally for seed germination under 16-h/8-h day/night for 20 days at 24°C. Twenty-day-old seedlings were transplanted onto new plates with full strength Murashige and Skoog (MS; Murashige and Skoog, 1962) medium pH 5.8 (Duchefa) containing 30 g/l sucrose, 0.5 μM NAA (Sigma-Aldrich), 5 μM N6-Benzylaminopurine (Duchefa), and 0.8% agar. The transplanted seedlings were grown under a growth cabinet with 16-h/8-h day/night at 25°C until they had thick hypocotyls, which took ∼3 months. *A. rhizogenes* transformation was performed only on seedlings with thick hypocotyls.

To prepare seedlings for transformation, they were incubated at 4°C in the dark for 4 days, before being pierced at the root–hypocotyl junction with a 0.4 mm × 20 mm syringe needle. Bacterial paste [*A. rhizogenes* strain AR1193 with pIV10 (empty vector) or with *DR5:GUS* grown on solid YEB medium (Van Larebeke et al., 1977) with antibiotics for 48 h] was directly applied onto the wound using a spatula. The treated seedlings were transferred to Petri dishes with 1/2 strength MS medium containing 100 µM acetosyringone, 19 g/l sucrose, and 0.8% micro agar (Duchefa). The plates were kept in the dark for 4 days for 25°C before being transferred to a growth cabinet with 16-h/8-h day/night at 25°C. After 2 weeks, transgenic roots appeared at the wound sites, and the plantlets were transferred to small pots containing a mixture of soil (Plugg och Såjord, Weibulls Trädgard AB, Hammenhog, Sweden), sand (Rådasand, Lidköping, Sweden; 0.4–0.6 mm), and autoclaved vermiculite. They were kept in a growth chamber with a 15-h photoperiod and day/night temperatures of 24°C/19°C, with a light intensity of 60–100 μE m^−2^ s^−1^ and 65% relative humidity under a lid for another 2 weeks before either harvesting of roots for GUS activity staining (36 root systems), or transfer to new pots containing the same soil mixture, but with *D. glomerata* nodules containing *Candidatus* Frankia datiscae Dg1 (Persson et al., 2015), which had been surface sterilized by incubation in 70% EtOH with 0.5% SDS for 5 min, followed by incubation in 1% sodium hypochlorite, 0.1% SDS for 7 min, followed by six washes with sterile ddH_2_O, before being ground in ddH_2_O and mixed with the soil. After infection, plants were watered once per week with 1/4 strength Hoagland’s medium pH 6.2 with doubled phosphate concentration containing 1 mM KNO_3_, otherwise with deionized water. Infection with *Frankia* was repeated after 2 weeks by spreading a suspension of surface-sterilized ground nodules over the soil. Nodules were harvested after the leaves of the infected plants had turned dark green, which took 2–3 months. Altogether, 10 nodulated root systems were stained for GUS activity.

### Histochemical Staining for β-Glucoronidase Activity

*M. truncatula* hairy roots were harvested before inoculation and at 3, 9, and 21 days postinoculation (dpi) with *S. meliloti*. *M. truncatula* hairy roots containing the fluorescent marker protein DsRed were selected with a SteREO Lumar.V12 stereomicroscope (Carl Zeiss, Jena, Germany) equipped with a 43 HE filter set (EX BP 550/25, EM BP 605/70). Afterwards, samples were fixed in 0.5% (w/v) paraformaldehyde in 100 mM potassium phosphate buffer (pH 7) on ice for 45 min under vacuum. Then, the material was rinsed with 100 mM potassium phosphate buffer and stained for GUS activity in GUS reaction buffer {100 mM potassium phosphate buffer (pH 7) containing 0.5 mM EDTA, 0.1% (v/v) Triton X-100, 0.5 mM K_3_[Fe(CN)_6_], 0.5 mM K_4_[Fe(CN)_6_], and 1 mM X-Gluc (5-bromo-4-chloro-3-indolyl β-D-glucuronide)} at 37°C overnight in the dark.

*D. glomerata* hairy roots were harvested and washed in GUS reaction buffer {1/4 strength SB buffer (12.5 mM PIPES, 1.25 mM MgSO_4_, 1.25 mM ethylene glycol tetraacetic acid, pH 6.9) containing 1 mM EDTA, 0.1% (v/v) Triton X-100 with 0.25 mM K_3_[Fe(CN)_6_]}. Then, the samples were transferred to GUS reaction buffer containing 1 mM X-Gluc, vacuum-infiltrated three times, each time for 5 min and incubated for 1–24 hat 37°C in the dark. Then, the stained samples were transferred to fixative solution (0.1 M sodium phosphate buffer pH 6.8, 3% paraformaldehyde, 0.1% Tween 20, 0.1% Triton-X-100) and incubated at 4°C overnight. Later, the root samples were rinsed twice with 0.1 M sodium phosphate buffer (pH 6.8) and embedded in 3% agarose.

*M. truncatula* roots were sectioned on a vibratome 1000 Plus (Intracel, Royston, UK) at 100 µm thickness. *D. glomerata* roots were sectioned on a Leica VT1000E vibratome at 50–100 μm thickness (Leica Biosystems, Wetzlar, Germany).

### Microscopy and Imaging

GUS-stained *M. truncatula* and *D. glomerata* roots were examined under an SZX9 stereomicroscope (Olympus, Tokyo, Japan) with a DMC-FZ5GN digital camera (Panasonic, Kadoma, Osaka, Japan) or a SteREO Lumar.V12 stereomicroscope (Carl Zeiss) with an Axiocam MRc5 digital microscope camera (Carl Zeiss). *M. truncatula* root sections were viewed with a Leica DMBL microscope (Leica Microsystems, Wetzlar, Germany) and documented with a SPOT RT slider CCD camera (Diagnostic Instruments, Sterling Heights, MI, US). *D. glomerata* sections were observed under an Axiovert 200 M microscope (Carl Zeiss) using bright field microscopy; results were documented using an Axiocam 506 color camera (Carl Zeiss).

### LC-MS Analysis of Auxins and Cytokinins

*M. truncatula* wild-type roots (∼5 cm from the root tip) and mature nodules were harvested in November–February, whereas *D. glomerata* wild-type roots (∼5 cm from the root tip), hairy roots, and mature nodules were harvested in June–October. Frozen plant material (50–200 mg of roots and nodules, respectively) was ground in liquid nitrogen. At least three biological and two technical replicates were analyzed; the exceptions were auxin in *D. glomerata* nodules and chickpea roots (two biological replicates).

For detailed analysis of cytokinins by liquid chromatography–mass spectrometry (LC-MS), *trans*-zeatin (tZ), *trans*-zeatin-7-glucoside (tZ7G), *trans*-zeatin-*O*-glucoside (tZOG), *trans*-zeatin-9-glucoside (tZ9G), *trans*-zeatin riboside *O*-glucoside (tZROG), *cis*-zeatin (cZ), *trans*-dehydrozeatin (tDHZ), *trans*-dehydrozeatin-riboside (tDZR), *trans*-zeatin riboside (tZR), and *trans*-*N* *^6^*-(Δ^2^-isopentenyl)adenine (tiP), and IAA were to be extracted and purified. Extraction was conducted with 1.25 ml of 80% (v/v) methanol. Samples were vortexed for 10 s; 4 µl of 5 ppm internal standard (deuterated mix) in 20% (v/v) methanol was added; then, the mixture was vortexed again for 10 s. The deuterium-labeled phytohormones used were [^2^H_3_]*t*DZR, [^2^H_6_]*t*iP, [^2^H_5_]*t*Z, [^2^H_5_]*t*ZOG, [^2^H_5_]*t*ZR, [^2^H_5_]*t*ZROG, [^2^H_6_]ABA, and [^2^H_5_]IAA (Olchemim Ltd, Olomouc, Czech Republic). The suspension was incubated at 4°C for 30 min, vortexed and centrifuged at 20,000 *g* at 4°C for 15 min. Afterwards, the supernatant was filtered through Chromafix C18 columns (Macherey-Nagel, Düren, Germany) to remove particles of a size above 45 µm. As described above, the pellet was resuspended in the same volume of extraction solvent, then the suspension was vortexed and centrifuged, and the supernatant was filtered. Extracts from two extractions were pooled together and concentrated using a speedvac centrifuge. One milliliter of 80% (v/v) methanol was added to dried samples, and then the samples were incubated in an ultrasonic waterbath for 8 min or until the pellet was dissolved. After that, the suspension was vortexed and filtered through syringe filters Chromafil PET-20/15 MS (Macherey-Nagel, Düren, Germany) and stored at −80°C prior to LC-MS analysis. Analyses were carried out on a high-performance liquid chromatography (HPLC)/MS system consisting of an Agilent 1100 Series HPLC (Agilent Technologies, Santa Clara, CA, USA) equipped with a microwell plate autosampler and a capillary pump and connected to an Agilent Ion Trap XCT Plus mass spectrometer (Agilent Technologies, Santa Clara, CA, USA) using an electrospray interface (ESI) as described in Großkinsky et al. (2014).

For detailed analysis of auxins by LC-MS/MS, the auxins IAA, IAA-alanine (IAA-Ala), IAA-aspartate (IAA-Asp), IAA-leucine (IAA-Leu)/IAA-isoleucine (IAA-Ile), IAA-phenylalanine (IAA-Phe), 4-chloro-IAA (4-Cl-IAA), IAA-tryptophan (IAA-Trp), IAA-valine (IAA-Val), indole-3-butyric acid (IBA), and PAA were extracted and purified. Sample preparation was carried out as described in Müller and Munné-Bosch (2011). The extracts were dried in a speedvac centrifuge with a vacuum cryopumping at 45°C overnight, filled with nitrogen gas, and stored at −80°C. Twenty nanograms of [^2^H_5_]IAA (Cambridge Isotopes Laboratory, MA, USA) was used as an internal standard in individual samples. Prior to injection, dried samples were resuspended in 80% (v/v) methanol and filtered through a 0.45-µm Nanosep MF centrifugal filtration column (Pall Life Sciences, NY, USA). LC-MS/MS was performed using an Agilent 6530 Accurate Mass LC-MS Q-TOF (Santa Clara, CA, USA) or a Thermo UPLC Q Exactive Plus Orbitrap LC-MS/MS system (Thermo Fisher Scientific, Waltham, MA, USA). Samples were subjected to ESI in the Jetstream interface in both positive and negative modes. Conditions in the positive modes were as follows: gas temperature, 250°C; drying gas, 5 L min^−1^; nebulizer, 30 psig; sheath gas temperature, 350°C and flowrate of 11 L min^−1^; capillary voltage, 2,500 V; nozzle voltage, 500 V; and fragmentor voltage, 138 V. Conditions in the negative mode were as follows: gas temperature, 300°C; drying gas, 9 L min^−1^; nebulizer, 25 psig; sheath gas temperature, 350°C and flowrate of 11 L min^−1^; capillary voltage, 3,000 V; nozzle voltage, 500 V; and fragmentor voltage, 140 V. Samples were injected (7 µl) onto an Agilent Zorbax Eclipse 1.8 µm XDB-C18 2.1 × 50 mm column. Solvent A consists of 0.1% aqueous formic acid, and solvent B consists of 90% methanol with 0.1% formic acid. Free auxins and conjugates were eluted with a linear gradient from 10 to 50% solvent B from 0 to 8 min, 50 to 70% solvent B from 8 to 12 min (then hold from 12 to 20 min), and 70 to 10% solvent B from 20 to 21 min (then hold from 21 to 30 min) at a flowrate of 200 µl min^−1^. The instrument was run in extended dynamic mode over a range of *m/z* 50–950 using targeted collision induced dissociation (CID; N_2_ collision gas supplied at 18 psi) MS/MS (3 spectra s^−1^). Analysis of data was performed using Agilent MassHunter software v 5.0. Authentic auxin standards (IAA-Phe, IAA-Leu, IAA-Val, IAA-Trp, 4-Cl-IAA; Olchemim Ltd, Olomouc, Czech Republic; IAA-Asp, IAA-Ala, IAA-Ile, IAA, IBA, PAA; Sigma, St. Louis, MO, USA) were used to determine elution times, collision energies, detection/quantification limits, and for absolute quantification.

## Results

### Auxin Contents in Roots and Nodules of *Medicago truncatula* and *Datisca glomerata*

**Figure 1:**
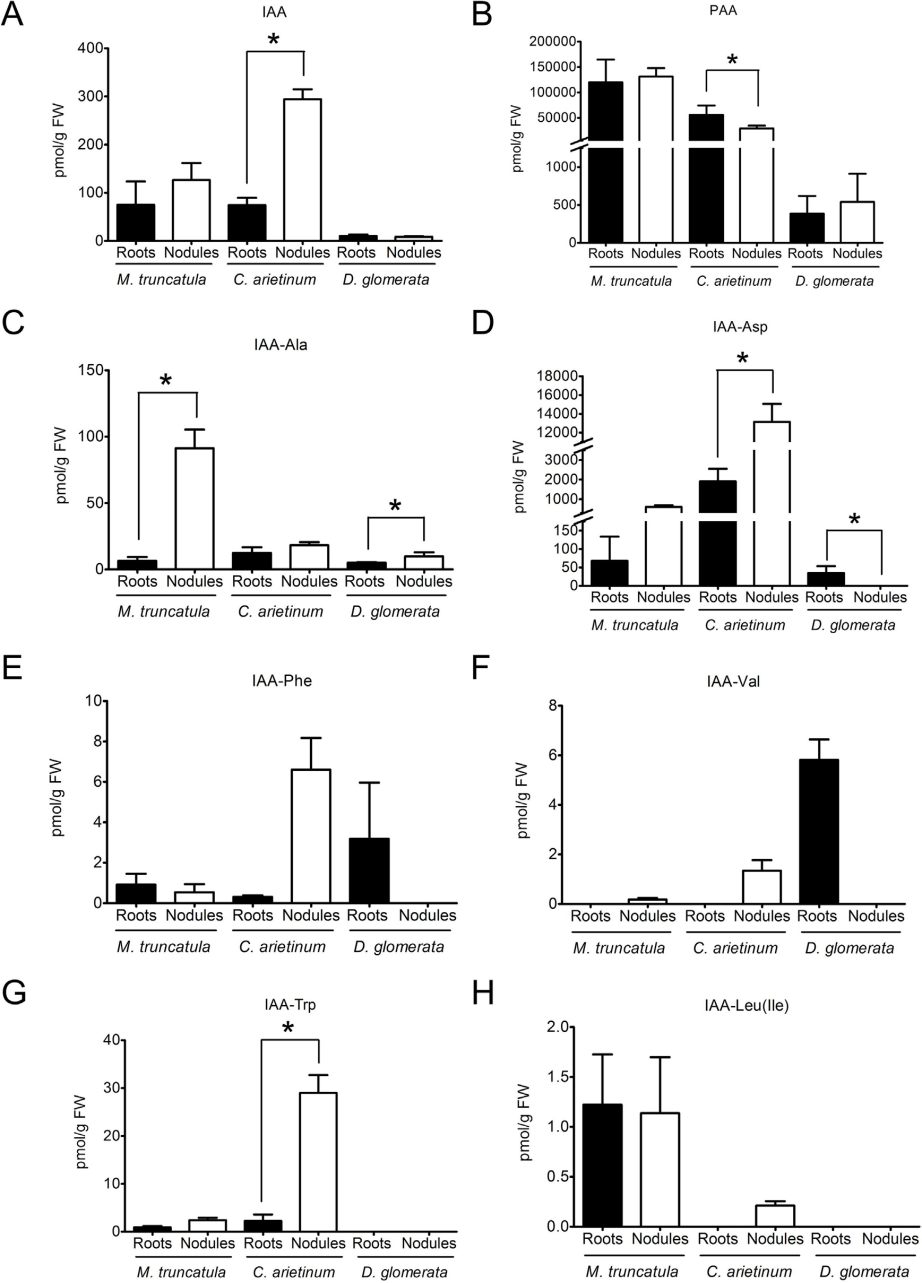
Auxin profiles of roots and nodules of the legumes *Medicago truncatula* and *Cicer arietinum* and the actinorhizal species *Datisca glomerata*.Endogenous concentrations of free and conjugated auxins were determined for roots (black) and nodules (white) of *M. truncatula*, *C. arietinum*, and *D. glomerata*. **(A)** indole-3-acetic acid; **(B)** phenylacetic acid; **(C)** IAA-alanine; **(D)** IAA-aspartate; **(E)** IAA-phenylalanine; **(F)** IAA-valine; **(G)** IAA-tryptophan; **(H)** IAA-leucine/IAA-isoleucine. Values represent means ± standard deviation, SD (n = 3). For each auxin/plant species, statistically significant differences between roots and nodules are labeled with an asterisk (Mann–Whitney *U* test, p ≤ 0.05). The numerical results are shown in **Supplementary Table S1**.

A detailed analysis of auxin composition and concentrations was performed for roots and nodules of *D. glomerata* and *M. truncatula*, using the extraction method described by Müller and Munné-Bosch (2011) followed by LC-MS/MS. Roots and nodules of a second legume, *C. arietinum* (chickpea), like *M. truncatula* a member of the inverted repeat-lacking clade, were included to assess the diversity of auxin patterns in different legumes. The results are depicted in **Figure 1**.

This method allowed the detection of the auxins IAA, IBA, 4-chloro-indole-3-acetic acid (4-Cl-IAA), and PAA and of the conjugated auxins IAA-Ala, IAA-Asp, IAA-Leu/IAA-Ile, IAA-Phe, IAA-Val, and IAA-Trp. Generally, IAA, IBA, 4-Cl-IAA, and PAA are considered as active auxins, while conjugated auxins are considered as inactive storage compounds (Korasick et al., 2013); it should be pointed out that conjugated forms of PAA were not analyzed as no standards were available. Concentrations of the auxins IBA and 4-Cl-IAA were below the limit of detection for the three plant species analyzed.

PAA, being present at levels much higher than those of IAA, turned out to be the dominant auxin in roots and nodules of both *M. truncatula* and *C. arietinum* as well as in roots and nodules of *D. glomerata* (**Figures 1A, B**). No significant differences were found between roots and nodules in the contents of PAA or IAA in both *M. truncatula* and *D. glomerata* (**Figures 1A, B**). However, this may not be representative of other legumes, as in chickpea we detected a significantly lower content of PAA and a significantly higher content of IAA in nodules than those in roots (**Figures 1A, B**). In *M. truncatula* and *D. glomerata*, the only conjugated auxins whose content was significantly higher in nodules than in roots was IAA-Ala , while IAA-Asp levels were only higher in nodules of *D. glomerata* (**Figures 1C, D**). Other auxin conjugates did not show any significant differences in their contents between roots and nodules in both *M. truncatula* and *D. glomerata* (**Figures 1E–H**). The results for chickpea were different: the contents of two conjugated auxins, IAA-Asp and IAA-Trp, were significantly higher in nodules than in roots. Absolute levels of active auxins differed between the three species examined.

In order to find out whether high PAA contents are typical for subterranean organs of actinorhizal plants or members of the Cucurbitales, roots from four other plant species were included in the analysis: *Casuarina glauca* (Casuarinaceae, Fagales) and *Coriaria myrtifolia* (Coriariaceae, Cucurbitales) as representatives of actinorhizal plants and *B. bowerae* and *Cucumis sativus* (cucumber) (Begoniaceae and Cucurbitaceae, both Cucurbitales) as non-symbiotic representatives of the Cucurbitales. The results are depicted in **Table 1**. PAA was the dominant auxin in roots of cucumber and *C. glauca*; however, no PAA could be detected in roots of *B. bowerae* and *C. myrtifolia*. Thus, high PAA levels in roots are neither a general feature of actinorhizal plants nor that of Cucurbitales. To see whether higher levels of PAA were associated with nodulation of determinate nodule-forming legumes, we analyzed the auxin profile of nodules of two more legumes, namely, *G. max* (soybean) and the model legume *L. japonicus* (**Table 1**). The results showed that PAA was indeed present in nodules of both species; however, due to high variability in PAA levels between biological replicates, no conclusion could be drawn regarding a possible role of PAA in nodulation of the determinate nodule-forming legumes.

**Table 1:** Root auxin profiles of two actinorhizal species and three members of the Cucurbitales; nodule auxin profiles of two legumes.

		Roots				Nodules	
		*Begonia bowerae*	*Casuarina glauca*	*Coriaria myrtifolia*	*Cucumis sativus*	*Lotus japonicas*	*Glycine max*
**free auxins**	**IAA**	3.8 ± 1.5	12.0 ± 3.0	4.2 ± 1.8	4.1 ± 2.3	66.9 ± 10.6	26.0 ± 11.6
	**IBA**	n.d.	1.0 ± 0.5	n.d.	n.d.	n.d.	2.7 ± 0.1
	**PAA**	n.d.	57.9 ± 19.6	n.d.	72.2 ± 15.3	460.0 ± 455.1	1,265.3 ± 1,458.2
	**4-Cl-IAA**	n.d.	1.3 ± 1.1	0.9 ± 0.8	n.d.	n.d.	n.d.
**conjugated auxins**	**IAA-Ala**	n.d.	1.3 ± 0.5	0.9 ± 0.9	5.1 ± 1.1	7.4 ± 1.8	20.8 ± 23.4
	**IAA-Asp**	17.1 ± 8.8	n.d.	5.8 ± 2.7	n.d.	2,084.3 ± 1,041.0	15.2 ± 9.0
	**IAA-Leu/IAA-Ile**	n.d.	n.d.	0.2 ± 0.1	n.d.	n.d.	n.d.
	**IAA-Phe**	n.d.	n.d.	1.8 ± 2.0	3.2 ± 2.2	n.d.	n.d.
	**IAA-Trp**	n.d.	n.d.	2.7 ± 1.8	3.9 ± 2.2	6.2 ± 4.5	n.d.
	**IAA-Val**	n.d.	0.5 ± 0.5	0.3 ± 0.2	n.d.	2.3 ± 1.2	2.1 ± 1.9

Root profiles were analyzed for four species; Casuarina glauca and Coriaria myrtifolia represent actinorhizal species, Begonia bowerae (Begoniaceae); Coriaria myrtifolia (Coriariaceae) and Cucumis sativus (Cucurbitaceae) are members of the Cucurbitales. Nodules of the legumes Lotus japonicus and Glycine max were analyzed as well. Average and standard deviation of six (C. glauca, C. myrtifolia) or three (B. bowerae, C. sativus, L. japonicus, G. max) biological samples are presented. Concentrations are given in pmol/g fresh weight.

### Cytokinin Contents in Roots and Nodules of *M. truncatula and D. glomerata*

**Figure 2:**
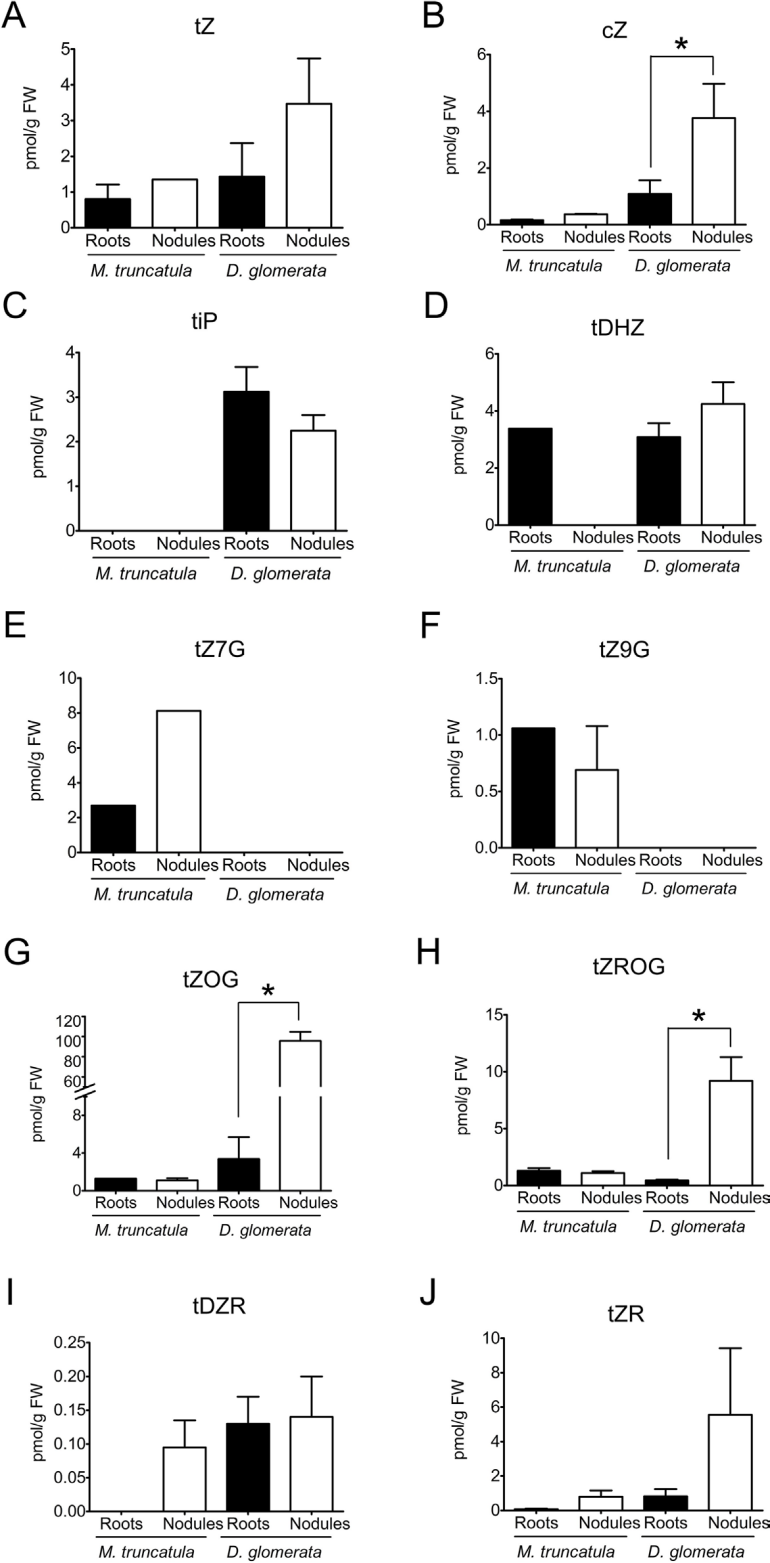
Cytokinins in roots and nodules of *Medicago truncatula* and *Datisca glomerata*. Endogenous concentrations of free and conjugated auxins were determined for roots (black) and nodules (white) of *M. truncatula* and *D. glomerata*. **(A)** *trans*-zeatin, **(B)** *cis*-zeatin; **(C)** *trans*-isopentenyladenine; **(D)** *trans*-dehydrozeatin; **(E)** *trans*-zeatin-7-glucoside; **(F)** *trans*-zeatin-9-glucoside; **(G)** *trans*-zeatin-O-glucoside; **(H)** *trans*-zeatin riboside O-glucoside; **(I)** tDZR—*trans*-dehydrozeatin-riboside; **(J)** *trans*-zeatin riboside. Values represent means ± SD (n = 3). Statistically significant differences between roots and nodules are labeled with an asterisk (Mann–Whitney *U* test, p ≤ 0.05). No significant different between roots and nodules were detected for *M. truncatula* (when no error bar is shown, the cytokinin was only quantifiable in one of the biological samples). The numerical results are shown in **Supplementary Table S1**.

As auxins typically act in concert with or antagonistically to cytokinins, the concentrations of cytokinins in roots and nodules were also quantified for both *M. truncatula* and *D. glomerata*. Endogenous concentrations of different cytokinins [*trans*-zeatin (tZ), *cis*-zeatin (cZ), *trans*-dehydrozeatin (tDHZ), *trans*-dehydrozeatin-riboside (tDZR), *trans*-zeatin riboside (tZR), and *trans*-*N*^6^-(Δ^2^-isopentenyl)adenine (tiP)] and their glycosylated forms [*trans*-zeatin-7-glucoside (tZ7G), *trans*-zeatin-9-glucoside (tZ9G), *trans*-zeatin-*O*-glucoside (tZOG), and *trans*-zeatin riboside *O*-glucoside (tZROG)] were determined for both *M. truncatula* and *D. glomerata* according to Großkinsky et al. (2014) (**Figure 2**). Generally, the glycosides are considered stable biologically inactive storage forms, while non-glycosylated forms are considered biologically active (Van der Krieken et al., 1990).

In *M. truncatula*, concentrations of cytokinins were close to the detection limit, and no significant differences could be detected between roots and nodules in the concentrations of individual cytokinins (**Figures 2 A–J**).

In *D. glomerata*, the total concentration of glycosylated cytokinins was much higher in nodules than in roots (Mann–Whitney *U* test, *p* ≤ 0.05); in particular, the concentrations of tZOG and tZROG were increased (**Figures 2G, H**). Thus, while in roots, the combined concentrations of non-glycosylated cytokinins (tZ, tDHZ, tDZR, cZ, and tiP) were higher than those of glycosylated cytokinins; in nodules, the combined concentrations of glycosylated cytokinins were significantly higher than those of non-glycosylated cytokinins. With regard to non-glycosylated cytokinins, the only detectable difference between roots and nodules was that concentrations of cZ were significantly elevated in nodules compared to those in roots (**Figure 2B**), although cZ is thought to play a lesser role in plant development than tZ and tiP do (Schäfer et al., 2015).

To conclude, *M. truncatula* roots and nodules did not differ significantly in the contents of active auxins. Levels of the inactive conjugated auxin IAA-Ala were significantly higher in nodules than in roots. Furthermore, *M. truncatula* roots and nodules did not differ significantly in the contents of non-glycosylated and glycosylated cytokinins, respectively. In *D. glomerata*, roots and nodules did not differ significantly in the contents of active auxins, whereas the contents of conjugated auxins in roots were slightly higher than those in nodules. As for cytokinins in *D. glomerata*, the contents of the glycosylated ZROG and tZOG and the non-glycosylated cytokinin cZ were significantly higher in nodules than in roots.

Altogether, auxin and cytokinins profiles of *M. truncatula* and *D. glomerata* showed some similarities. Roots and nodules in both species did not differ significantly in the contents of active auxins and non-glycosylated cytokinins, respectively. Based on concentrations of detected active auxins (IAA and PAA) and indisputably active cytokinins (tZ and tiP), auxin/cytokinin ratios were calculated for roots and nodules of both *M. truncatula* and *D. glomerata*. In *M. truncatula*, auxin/cytokinin ratios were 124 and 97 for roots and nodules, respectively, whereas in *D. glomerata* the ratios were 101 and 96, respectively.

### Distribution of Auxin Response Maxima in Transgenic Roots of Composite *M. truncatula* and *D. glomerata* Plants

**Figure 3:**
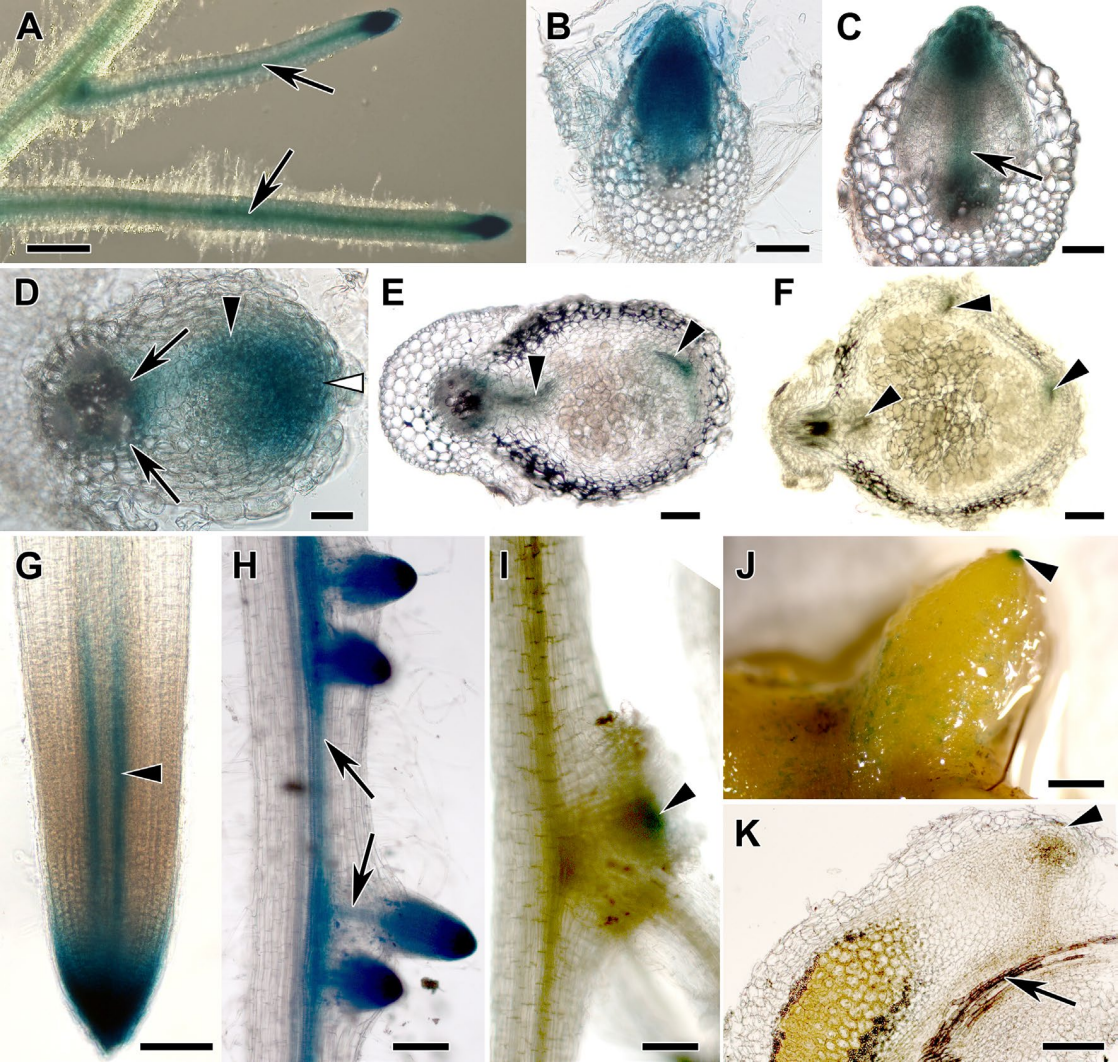
Transgenic hairy root systems expressing a *DR5:GUS* fusion. Light micrographs of **(A)** transgenic roots of composite *Medicago truncatula* plants, **(B–C)** root sections, and **(D–F)** nodule sections and **(G–I)** transgenic roots of composite *Datisca glomerata* plants and **(J)** a nodule and **(K)** a nodule section are shown. **(A)** In *M. truncatula* roots, GUS activity was detected in the root tips, lateral root primordia, vascular bundles, and at lower levels in the root cortex. **(B)** During lateral root formation, GUS activity was observed in the lateral root primordia, and **(C)** lower activity was found in the lateral root vascular system during lateral root emergence (arrow). **(D)** During nodule development, GUS activity was localized in the nodule primordium (black arrowhead) and in the incipient nodule meristem (white arrowhead) as well as at the nodule base (arrows) at 4 dpi. **(E)** In differentiated nodules at 9 dpi, GUS activity was present in the nodule meristem and in the nodule vascular system (black arrowheads). **(F)** At 3 wpi, no GUS activity could be detected in the nodule anymore (black arrowheads point at the nodule vascular system).In transgenic roots of composite *D. glomerata* plants, **(G)** GUS staining was restricted to the root tip and to the peripheral part of the xylem (arrowhead). **(H)** GUS staining was found in lateral root primordia; during lateral root development, it became restricted to the root tip and the vascular system (arrows). **(I)** Nodule primordia—distinguishable from lateral root primordia since they are flatter—also showed GUS staining (arrowhead points at a nodule primordium that is positioned at the base of a lateral root). **(J)** Mature nodules only showed GUS staining at the tips of actively growing lobes (arrowhead), the weak staining on parts of the periderm represents epiphytic bacteria with endogenous GUS activity. **(K)** Analysis of longitudinal sections showed that GUS activity was restricted to a cell layer distal of the meristem of the nodule lobe (arrowhead). No *DR5:GUS*-dependent GUS staining was ever detected associated with a *D. glomerata* nodule vascular bundle (an arrow points at the nodule vascular bundle in K). Size bars: **(A)**, 1 mm; **(B)** and **(C)**, 100 µm; **(D)**, 50 µm; **(E)**, 100 µm; **(F)**, 200 µm; **(G)**, 100 µm; **(H)**, 200 µm; **(I)**, 100 µm; **(J)**, 1 mm; **(K)**, 200 µm.

The synthetic auxin-responsive promoter *DR5* (Ulmasov et al., 1997) was used to monitor the auxin response in transgenic roots of composite *M. truncatula* plants. The activity of this promoter had been shown to correlate with the sites of auxin accumulation in *A. thaliana* roots (Casimiro et al., 2001). In non-inoculated transgenic roots, strong *DR5* promoter activity as signified by GUS staining was detected in root tips and lateral root primordia, while lower activities were found in the vascular system. Activities in the root cortex were very low (**Figures 3A–C**). After inoculation with *S. meliloti*, GUS activity was found in nodule primordia at 4 days postinoculation (dpi; **Figure 3D**) and in the tip of differentiating nodules at 9 dpi (**Figure 3E**). In mature, 3-week-old nodules, GUS staining had more or less disappeared (**Figure 3F**).

Transgenic roots of composite *D. glomerata* plants were obtained by transformation with *A. rhizogenes* carrying the integration vector pIV10 with a *DR5:GUS* fusion construct. pIV10 without insert showed no background activity in transgenic roots of *D. glomerata* (data not shown). Strong *DR5* promoter activity was detected in the root tip (**Figure 3G**) and in lateral root primordia, while lower activities were found in the vascular system (**Figure 3H**). No *DR5* promoter activity was detected in the root cortex. The root tip staining was due to strong promoter activity in the root cap (**Figure 3G**). GUS activity was also detected in nodule primordia (**Figure 3I**). In nodules, GUS activity was confined to a single-cell layer distal to the nodule lobe meristem, i.e., to the nodule phellogen covering the nodule apex (**Figure 3J** shows the whole nodule, **Figure 3K** shows a nodule section).

### Response of *M. truncatula* and *D. glomerata* Roots to Exogenous Auxin

**Figure 4:**
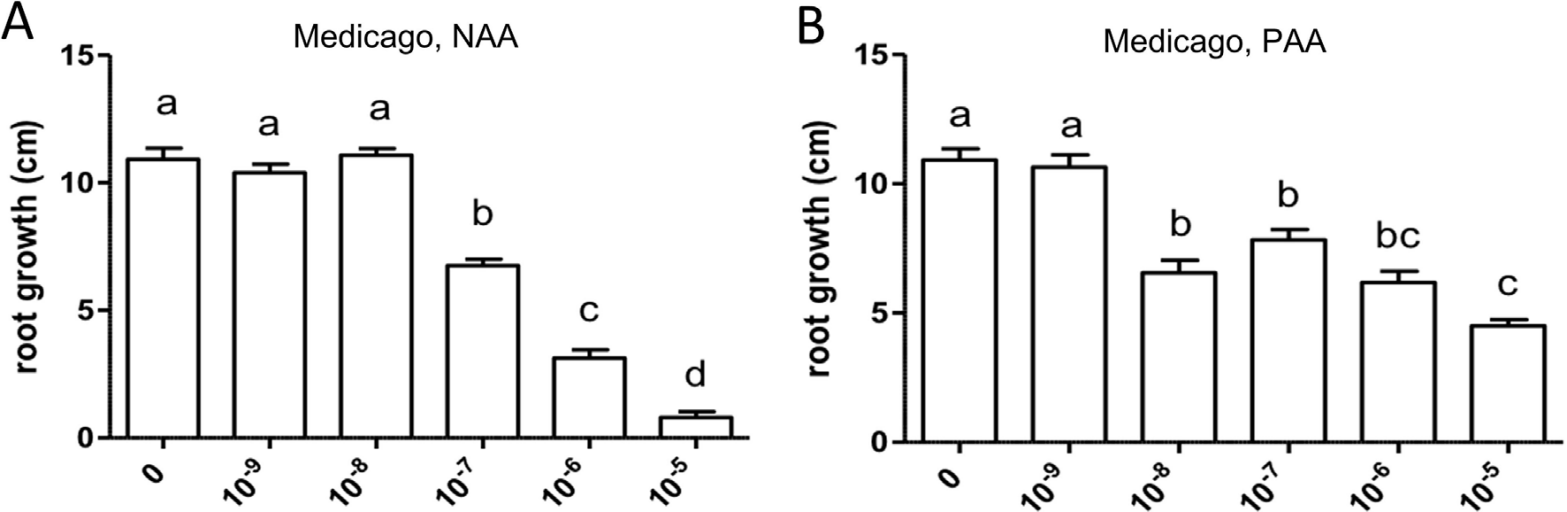
Effect of NAA **(A)** and PAA **(B)** on average main root length (cm) in *Medicago truncatula*. Values represent means ± SEM. One-way ANOVA with Tukey’s *post hoc* test was used to assess significant differences between treatment groups. Values labeled with different letters are significantly different (*p* ≤ 0.05). *n* = 9–12.

The cell response to auxin in *M. truncatula* roots was studied by application of the synthetic auxin NAA and the natural auxin PAA, respectively, to seedlings in axenic culture at the following concentrations: 10^−9^, 10^−8^, 10^−7^, 10^−6^, and 10^−5^ M. Some pictures of plant growth under the experimental conditions are shown in **Supplementary Figure S1**. These experiments were performed three times for main root length growth and two times for root branching. Both auxins had an inhibitory effect on the elongation of the primary root, with different sensitivity (**Figure 4**). In this experiment, sensitivity to PAA (10^−8^ M) was higher than sensitivity to NAA (10^−7^ M); however, in two repetitions of the experiment, sensitivity to PAA was lower than sensitivity to NAA.

**Figure 5:**
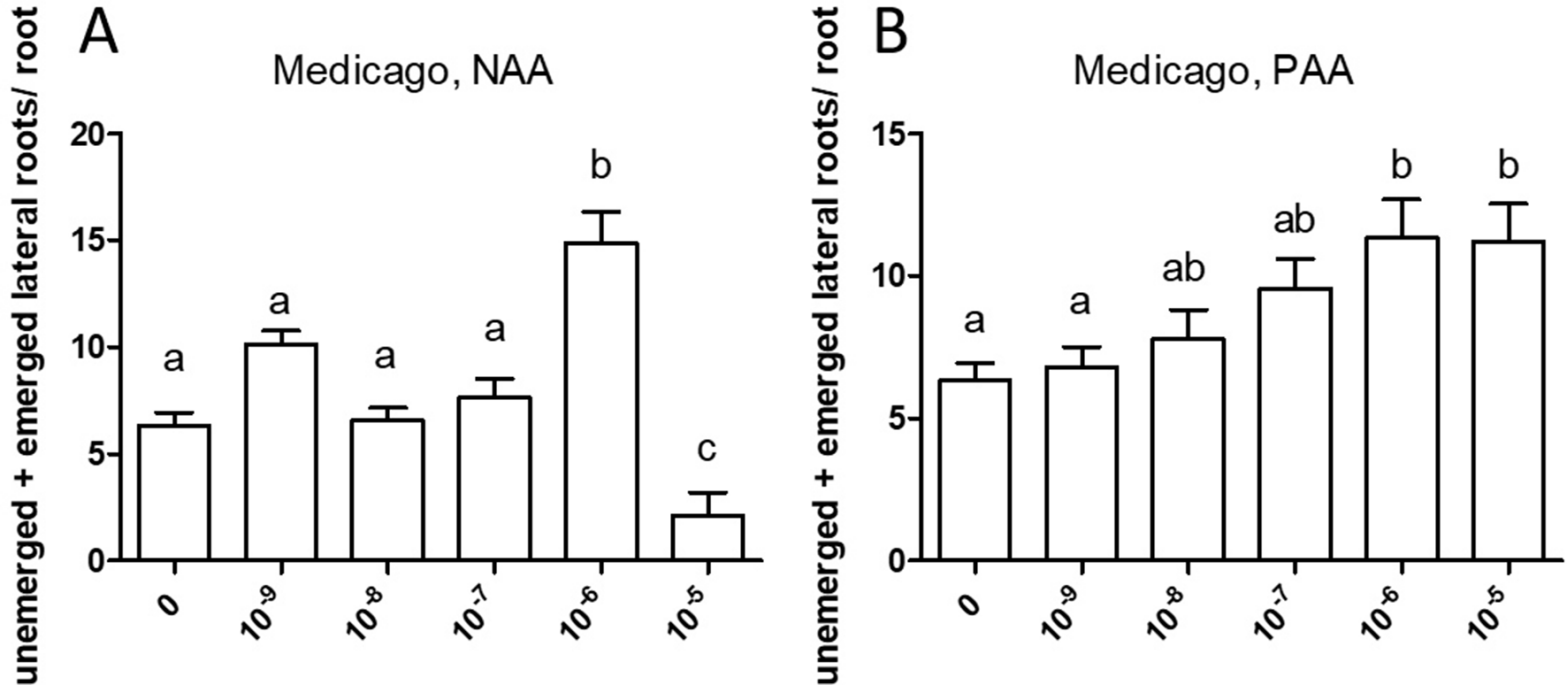
Effect of NAA **(A)** and PAA **(B)** on root branching in *Medicago truncatula*. The figure shows the number of emerged and unemerged lateral roots (primordia) per main root. Values represent means ± SEM. One-way ANOVA with Tukey’s *post hoc* test was used to assess significant differences between treatment groups. Values labeled with different letters are significantly different (p ≤ 0.05). n = 9–12.

**Figure 6:**
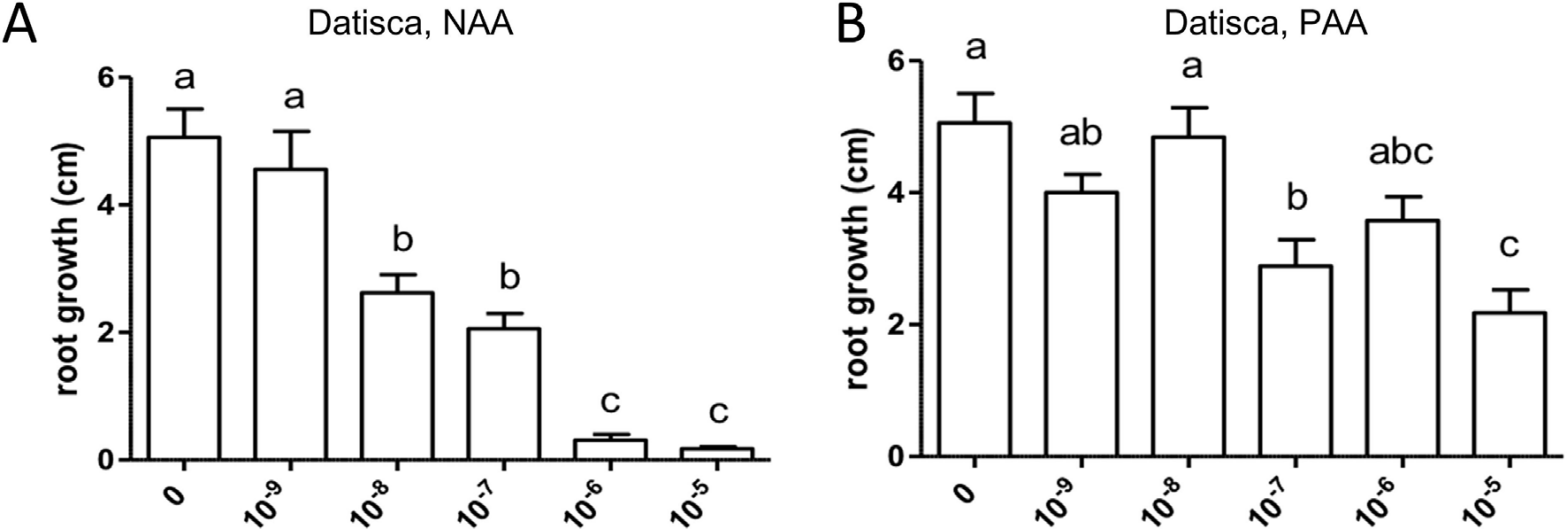
Effect of NAA **(A)** and PAA **(B)** on average main root length (cm) in *Datisca glomerata*. Values represent means ± SEM. One-way ANOVA with Tukey’s *post hoc* test was used to assess significant differences between treatment groups. Values labeled with different letters are significantly different (p ≤ 0.05). n = 9–12.

**Figure 7:**
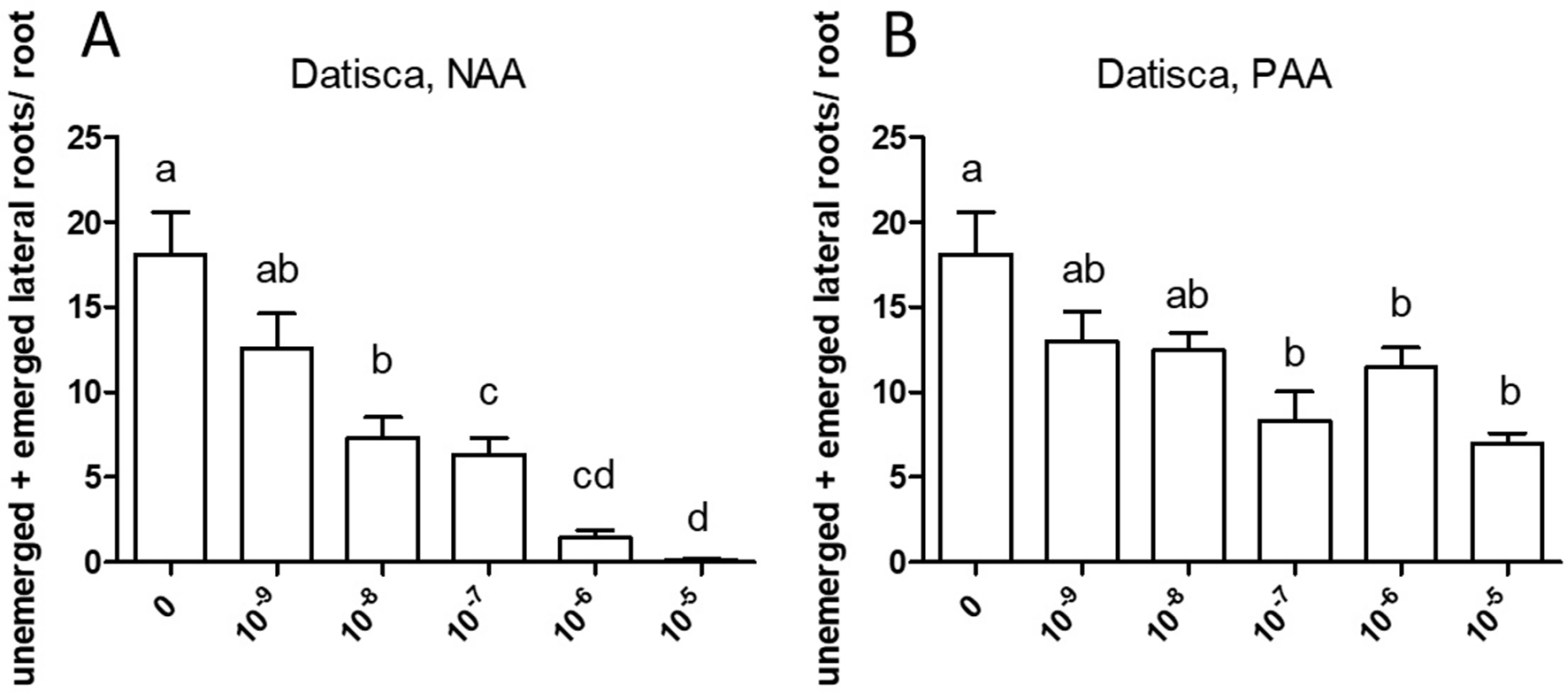
Effect of NAA **(A)** and PAA **(B)** on branching of the seedling root of *Datisca glomerata*. The figure shows the number of emerged and unemerged lateral roots (primordia) per main root. Values represent means ± SEM. One-way ANOVA with Tukey’s *post hoc* test was used to assess significant differences between treatment groups. Values labeled with different letters are significantly different (p ≤ 0.05). n = 9–12.

Similarly, both auxins enhanced root branching, as evaluated by measuring the numbers of emerged and unemerged lateral roots (lateral root primordia) per main root (**Figure 5**). In this series, sensitivity to NAA was equal to the sensitivity to PAA (10^−6^ M). The optimum curve of the auxin effect on lateral root branching was visible for NAA in that root branching at 10^−5^ M (**Figure 5B**) was lower than at control levels (**Figure 5A**), but not for PAA: root branching at 10^−5^ M was the same as at 10^−6^ M (**Figure 5B**). Concentrations higher than 10^−5^ M were not used because, already at 10^−5^ M, the effect on root length growth was so strong (**Figure 4**) that counting of primordia was very difficult. Similar results were obtained by measuring the number of emerged lateral roots only (**Supplementary Figure S2**). As for *M. truncatula*, the auxin response was studied in *D. glomerata* seedlings using NAA and PAA, respectively, at the concentrations 10^−9^, 10^−8^, 10^−7^, 10^−6^, and 10^−5^ M. The effect on the growth of the main root was similar to that in *M. truncatula* in that an inhibitory effect was observed at 10^−8^ M for NAA and at 10^−7^ for PAA, respectively (**Figure 6**). However, in contrast to *M. truncatula*, neither auxin promoted root branching at any concentration examined. On the contrary, NAA inhibited root branching (expressed as lateral roots and lateral root primordia per main root) starting at 10^−8^ M and PAA at 10^−7^ M (**Figure 7**). With NAA concentrations above 10^−8^ M, the inhibitory effect increased, while with PAA concentrations above 10^−7^ M, the inhibitory effect remained the same. Similar results were obtained by measuring the number of emerged lateral roots per main root only (**Supplementary Figure S2**).

### Adventitious Root Formation in *D. glomerata* in Response to Exogenous Auxin

**Figure 8:**
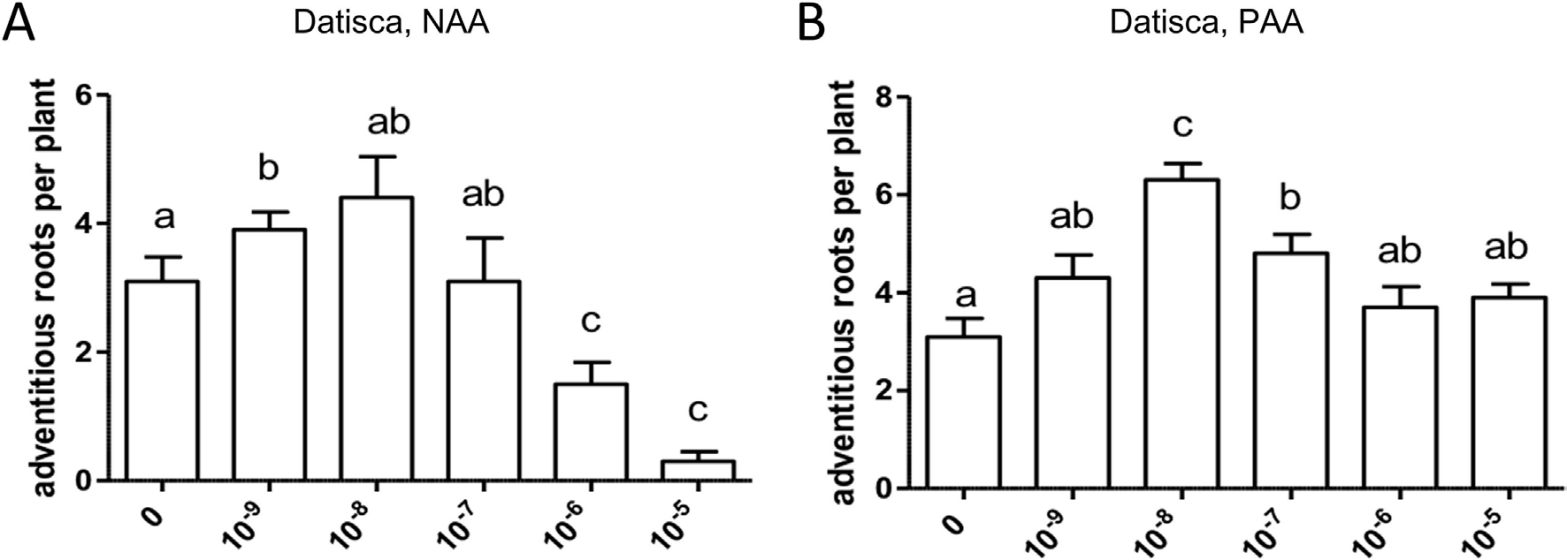
Effect of NAA **(A)** and PAA **(B)** on adventitious root formation in *Datisca glomerata*. Values represent means ± SEM. One-way ANOVA with Tukey’s *post hoc* test was used to assess significant differences between treatment groups. Values labeled with different letters are significantly different (p ≤ 0.05). n = 9–12.

Adventitious roots are formed from stem or leaf-derived cells. Development of adventitious roots is a complex process that is affected by multiple factors including phytohormones, e.g., auxin (Steffens and Rasmussen, 2016), light, nutritional status, and stress responses such as wounding (Geiss et al., 2009). The effect of auxin on adventitious root formation requires polar transport (Vidoz et al., 2010; Sun et al., 2015). In the growth system used in this study, *D. glomerata* forms adventitious roots (**Supplementary Figure S3**), while *M. truncatula* does not. Adventitious root formation was not restricted to the hypocotyl or a definable part of the stem. The effect of exogenously applied auxin on adventitious root formation was evaluated for *D. glomerata* (**Figure 8**). Application of NAA or PAA, respectively, increased adventitious root formation following an optimum curve, and the induction of the morphogenetic program was more sensitive to NAA than to PAA. Superoptimal NAA concentrations (10^−6^–10^−5^ M) reduced adventitious root formation to values below those in the control, while superoptimal PAA concentrations reduced it to control values.

## Discussion

To the best of our knowledge, the results of this study represent the first comparison of quantifications of endogenous concentrations of auxins and cytokinins in roots as well as nodules of the legume *Medicago truncatula* and the actinorhizal plant *Datisca glomerata*. Concentrations of auxins and cytokinins have been compared for roots and nodules of other leguminous and actinorhizal species (Silver et al., 1966; Dullaart, 1970; Henson and Wheeler, 1976; Wheeler et al., 1979; Mansour, 1994; Perrine-Walker et al., 2010); nevertheless, many of the previous quantifications were based on bioassays, which do not provide results that can be directly compared with the state of the art LC-MS/MS data obtained in our studies.

Similar levels of IAA were found in nodules and roots of the legume *M. truncatula*. This result is different from bioassay-based determinations of IAA performed for other legumes, such as chickpea, and actinorhizal plants, i.e., *Vicia faba*, *Lupinus luteus*, and *Alnus glutinosa* (Dullaart, 1970; Wheeler et al., 1979). Thus, either relative IAA levels in roots organs depend on the species, or the bioassays did not detect, or did not show the same sensitivity for all auxins. In the actinorhizal species *D. glomerata*, IAA levels in roots and nodules were close to the detection limit. Notably, the levels of some IAA conjugates did differ significantly between roots and nodules, with a higher level of IAA-Ala in nodules than in roots in both *M. truncatula* and *D. glomerata* and a higher level of IAA-Asp and IAA-Trp in nodules than in roots of chickpea. It might be interesting to investigate in the future whether these conjugates play any active role in addition to free IAA or whether they just reflect continuous adjustment of free IAA levels through temporary storage.

Ever since PAA had been tested as a synthetic auxin in 1935 (Haagen-Smit and Went, 1935), it was known to have auxin-like effects. However, it was noted already in 1935 that PAA was not transported in a polar manner like IAA, which was supported by Suttle and Mansager (1986) and further confirmed by Sugawara et al. (2015), who showed that NPA, an inhibitor of basipetal IAA transport, does not affect PAA transport in maize coleoptiles. Morris and Johnson (1987) reported that PAA inhibited the polar transport of IAA in intact plants and stem segments of pea and proposed this mechanism as basis for an indirect role in growth regulation by PAA. The synthesis of PAA follows another pathway than that of IAA (Cook et al., 2016), which would allow different regulatory mechanisms. Nevertheless, PAA plays an important role in several aspects of plant growth and development, upregulating the same early auxin-responsive genes as IAA (Sugawara et al., 2015).

In this study, high PAA levels were found in nodules from the legumes *M. truncatula*, *Cicer arietinum*, *Lotus japonicus*, and soybean and in roots and nodules of the actinorhizal plant *D. glomerata*. Previously, significant PAA levels had been reported for roots and nodules of the actinorhizal species *Casuarina glauca* (Perrine-Walker et al., 2010). Furthermore, PAA has been detected immunologically in infected cells of nodules of the actinorhizal plant *Discaria trinervis* (Rhamnaceae, Rosales; Imanishi et al., 2014). The presence of high PAA levels in *C. glauca* roots was confirmed in this study using seeds from New Zealand. The ratio of IAA and PAA in roots of *C. glauca* from New Zealand was different from that published previously, indicating that the composition of the active auxin fraction in roots differs between ecotypes. Thus, the presence of PAA as such did not seem to be an ecotype-dependent effect. Analysis of the roots of the actinorhizal plant *Coriaria myrtifolia* (Coriariaceae, Cucurbitales) showed that PAA is not present in roots of all actinorhizal plants; further analysis of roots of two non-symbiotic members of the Cucurbitales showed that only one of them (cucumber) contained PAA while the other one (*B. bowerae*) did not. Thus, the presence of PAA in roots is not correlated with the ability to nodulate. Unfortunately, conjugated forms of PAA could not be analyzed as no standards were available. In all systems examined, PAA, when present at all, was present at significantly higher levels than IAA (**Figure 1**; **Table 1**). Altogether, we found PAA in all nodules analyzed, and it was the auxin found at the highest concentrations in nodules of all legumes as well as *D. glomerata*.

Exogenously supplied PAA showed an effect on root growth similar to those achieved by NAA. In most cases, sensitivity of root growth and root branching to NAA was one to two orders of magnitude higher than that to PAA in both *M. truncatula* and *D. glomerata*. At this point, it should be mentioned that the results were confirmed in several independent experiments, and while the results were the same qualitatively (primary root growth was inhibited in all cases, and the effect of auxins on root branching differed in both species), the sensitivity to NAA and/or PAA could shift. This might be due to the fact that the roots were growing on top of agar plates and not in liquid culture with equal distribution of the phytohormone on all sides (Dubrovsky and Forde, 2012).

The fact that high, variable levels of PAA were found in nodules of three actinorhizal species (Perrine-Walker et al., 2010; Imanishi et al., 2014 and this study) and four legumes (this study) suggests that PAA might play a role in root nodule symbioses (**Figure 1**; **Table 1**). This suggestion was further supported by the fact that a number of *Frankia* strains infecting *A. glutinosa*, *Elaeagnus angustifolia* (Hammad et al., 2003), and *C. glauca* (Perrine-Walker et al., 2010) were shown to produce PAA in culture, suggesting that the microsymbionts could contribute to the PAA contents of nodules. In this context, it is interesting that homologues of the genes that encode enzymes of the PAA biosynthetic pathway in the *Casuarina*-infective *Frankia* strain CcI3 (Perrine-Walker et al., 2010) are present in the two sequenced genomes of *D. glomerata*-nodulating *Frankia* strains, *Candidatus* Frankia datiscae Dg1 (Persson et al., 2015), and *Candidatus* Frankia californiensis Dg2 (Nguyen et al., 2016; Normand et al., 2017). These data suggest that Dg1, as well as CcI3, can contribute to the accumulation of PAA in *Frankia*-infected nodule cells given the fact that PAA can pass the bacterial membrane and the perisymbiotic membrane by passive diffusion (Perrine-Walker et al., 2010; Simon and Petrášek, 2011). However, this would not agree with the fact that PAA concentrations in non-inoculated *D. glomerata* roots were comparable to those in nodules; yet, this fact might be explained by the assumption that, in nodules, excessive amounts of PAA could be inactivated by conjugation. Altogether, it is still unclear whether the microsymbionts contribute to the PAA contents of actinorhizal (or legume) nodules.

In *M. truncatula*, concentrations of cytokinins determined in roots and nodules were close to the detection limit; therefore, no conclusion can be drawn regarding cytokinin distribution in the root systems of this plant species. While high cytokinin responses have been found in the infected zone of nodules of soybean (Fisher et al., 2018), we did not detect higher levels of active cytokinins in *M. truncatula* nodules compared to roots. However, it is possible that local differences in cytokinin (and auxin) concentrations exist inside nodules that we could not resolve in our study. In *D. glomerata*, higher concentrations of some cytokinins in nodules than in roots are consistent with the bioassay-based quantifications for other actinorhizal species, i.e., *A. glutinosa*, *Myrica gale* (Fagales), and *Purshia tridentata* (Rosales; Wheeler et al., 1979). In *D. glomerata*, the biologically inactive storage form tZOG constituted a large portion of cytokinins in nodules. This is consistent with the results obtained for *A. glutinosa* nodules by Wheeler et al. (1979), which were based on differential extraction, paper chromatography, and bioassays, and led to the conclusion that most of the nodule cytokinins were present as glycosylated conjugates. Thus, since a large portion of the cytokinins found in *D. glomerata* nodules was present in the glycosylated storage form, and therefore probably was inactive, it seems that *D. glomerata* nodules, similar to *A. glutinosa* nodules, tend to keep cytokinins converted to the storage forms. This finding indicates that the regulation of cytokinin homeostasis involves not only *de novo* synthesis and degradation but also the reversible inactivation, supporting the need for a finely balanced nature of cytokinin metabolism. It was interesting that the level of the biological active *trans*-zeatin (tZ) was significantly higher in nodules than in roots. This is in agreement with mainly genetic evidence for a role of zeatin-type cytokinins early in legume nodule organogenesis and development (Reid et al., 2017; Mens et al., 2018). The fact that root-specific auxin/cytokinin ratios are not suited to distinguish nodulating from non-nodulating species is in agreement with the need for a concerted action of auxin and cytokinin for root cortical cell division and differentiation in general, both in response to rhizobia and also in response to other beneficial microbes and pathogens (Boivin et al., 2016).

The auxin distribution in roots and nodules of composite transgenic plants of both *M. truncatula* and *D. glomerata* was studied using the synthetic auxin-responsive promoter *DR5* fused to the β-glucuronidase reporter gene. In non-inoculated roots of composite plants of both species, activity of the promoter was detected in root tips and lateral root primordia as well as in the protoxylem, similar to previous reports (Herrbach et al., 2014; Fisher et al., 2018). Additionally, the *DR5* promoter was active in nodule primordia of both species; however, in *D. glomerata*, this activity was only found in very young primordia. At later stages of *M. truncatula* nodule development, promoter activity was associated with the nodule tip containing the meristem, while staining associated with the nodule vascular bundles was gradually disappearing, in agreement with other reports from *M. truncatula* and *Trifolium repens* (Mathesius et al., 1998; Breakspear et al., 2014; Franssen et al., 2015). In young and mature *D. glomerata* nodules, *DR5:GUS* expression was confined to the tips of nodule lobes, to a single-cell layer on top of the nodule lobe meristem. While *DR5:GUS* expression is only representing local auxin maxima, it was still surprising that only such a low number of cells expressed *DR5:GUS*, particularly in view of the fact that *DR5:GUS* expression had been shown to be induced by PAA in *Arabidopsis* (Sugawara et al., 2015). In this context, it should be noted that, in *C. glauca*, *DR5:GUS* was not expressed in the infected cells of nodules, although direct immunological localization of IAA and PAA showed that IAA and PAA are accumulated in infected nodule cells (Perrine-Walker et al., 2010). Similarly, a study on the intercellularly infected actinorhizal species *D. trinervis* (Rhamnaceae, Rosales) showed that PAA could be immunolocalized in infected cells of root nodules, while a *DR5:Venus-NLS* construct was expressed in the meristematic region of the nodule but not in the infected nodule cells (Imanishi et al., 2014). Thus, it is likely that also infected nodule cells of *D. glomerata* accumulate auxin, but that the auxin signal transduction pathway is not able to activate the synthetic *DR5* promoter in infected cells of actinorhizal nodules.

The most striking result of this study came from the comparison of auxin effects on root development in *M. truncatula vs. D. glomerata*. First, the activity of PAA was similar to that of NAA in both species, and sensitivities to both auxins were similar. There was one consistent difference between the response to NAA *vs.* PAA, namely, the response to superoptimal concentrations; this might be explained by the assumption that the optimum curve of the PAA response is broader than the optimum curve of the NAA response. For *M. truncatula*, the exogenous application of both auxin species to roots resulted in the classic positive effect on root branching. Owing to the concurrent effect on primary root growth, concentrations higher than 10^−5^ M were not examined. For *D. glomerata*, however, both auxin species had a purely negative effect on root branching. Interestingly, with regard to the formation of adventitious roots, a phenotype that could only be examined for *D. glomerata* as *M. truncatula* did not form any adventitious roots in our growth system; both auxins had a positive effect in an optimum curve. In this context, it is important to point out that both adventitious root formation and lateral root initiation are supposed to require polar auxin transport (Sukumar et al., 2013; Verstraeten et al., 2014).

A similar negative effect of auxin on root development had been observed for NAA on *Cucurbita pepo* (Cucurbitaceae; Ilina et al., 2018). However, *Cucurbita* spp. are characterized by a special type of root branching, where lateral root primordia are initiated in the meristematic zone of the parental root. The negative auxin effect on root branching here might be attributed to the fact that the lateral root primordia appear in an area where the auxin concentration is already very high, which could prevent an effect of exogenous auxin (Ilina et al., 2018). Alternatively, the negative auxin effect could be a result of the distribution of auxin transporters: experiments on the distribution of exogenously applied fluorescent auxin analogues in *Arabidopsis* seedling roots showed that the auxin analogues did not accumulate in the meristematic zone but in the root cap and the elongation zone (Hayashi et al., 2014). Yet, *D. glomerata* clearly does not form lateral root primordia in the meristematic zone; otherwise, the corresponding auxin response maxima would be visible on **Figure 3G**. Hence, root branching mechanisms of *D. glomerata* deserve further investigation.

All root nodules are lateral root organs; yet, legume nodules differ from nodules of all other symbiotic plants in that the legume nodules have a peripheral vascular system and infected cells are located in the central nodule tissue (Mylona et al., 1995). Our study shows that the ratio of active auxin/cytokinin remained more or less the same between roots and nodules in *M. truncatula* as well as in *D. glomerata*, although it remains to be shown which auxins and cytokinins are biologically active in which tissue. Furthermore, no auxin response has ever been detected in the vascular tissue of *D. glomerata* nodules using *DR5:GUS*, while the auxin response in the vascular system of *M. truncatula* nodules is present over a long period in nodule development, and this also occurs in determinate nodules (see, e.g., Takanashi et al., 2011; Fisher et al., 2018). This indicates that either the auxin signal transduction pathway that activates the *DR5* promoter is not sufficiently active in the vascular system of actinorhizal nodules or that, in spite of the common evolutionary origin of both symbioses, cell responses to phytohormones differ significantly between nodules and roots in legumes *vs.* actinorhizal plants.

## Conclusions

Auxin and cytokinin profiles of *M. truncatula* and *D. glomerata* showed some similarities. Roots and nodules in both species did not differ significantly in the contents of active auxins and non-glycosylated cytokinins, respectively.

PAA was the dominant auxin in all types of examined root nodules: nodules of *M. truncatula*, *C. arietinum*, *L. japonicus*, soybean, and *D. glomerata*. It was also the dominant auxin in roots of *M. truncatula* and *D. glomerata*. For both roots and nodules, the concentrations of PAA and IAA were higher in *M. truncatula* than those in *D. glomerata*.

The levels of *cis*-zeatin and of the glycosylated cytokinins tZOG and tZROG were much higher in nodules than in roots, but ratios of active auxins to active cytokinins were similar between roots and nodules and similar between *M. truncatula* and *D. glomerata.* Roots of *M. truncatula* and *D. glomerata* also showed certain similar responses to exogenous application of NAA and PAA, respectively and roots of both species were more sensitive to NAA. In both species, auxins inhibited the growth of the primary root. However, while in *M. truncatula* auxins increased root branching, in *D. glomerata* they inhibited root branching but promoted adventitious root formation.

In *M. truncatula* nodules, *DR5:GUS* was expressed in the meristematic region, while expression in the vascular system declined in the course of nodule development. In *D. glomerata* nodules, expression was confined to a single-cell layer distal to the nodule lobe meristem. In combination with the data on auxin levels, these results suggest that the auxin signal transduction pathway that activates the *DR5* promoter is not sufficiently active in all cell types of actinorhizal nodules to activate *DR5* expression.

## Data Availability

All datasets generated for this study are included in the manuscript and the supplementary files.

## Author Contributions

Conceptualization: ID, UM, and KP; methodology: ID, PM, JN, TR, KD, UM, and KP; investigation: ID, PM, AN, EG, JN and UM; formal analysis: ID, JN, PM, EG, and UM; visualization: ID, AN, PM, KD, KP, and UM; writing—original draft: ID, KD, UM, and KP; writing—review and editing: ID, PM, JN, TR, KD, UM, and KP; funding acquisition: UM, TR, and KP; supervision: UM and KP.

## Funding

This study was supported by two grants from the Swedish Research Council Vetenskapsrådet (VR 2007-17840-52674-16 and VR 2012-03061) and by a grant from Carl Tryggers Stiftelse för Vetenskaplig Forskning (CTS 13:354) to KP, by a grant from the Russian Science Foundation (analyses of auxin response pattern, grant no. 16-16-00089) to KND, and by a grant from the Ministry of Education, Youth and Sports of CR within the National Sustainability Program I (NPUI, grant number LO1415) to TR. UM was supported by the Australian Research Council (DP150102002).

## Conflict of Interest Statement

The authors declare that the research was conducted in the absence of any commercial or financial relationships that could be construed as a potential conflict of interest.

## Abbreviations

Ala, alanine; Asp, aspartate; cZ, *cis*-zeatin; 4-Cl-IAA, 4-chloro-indole-3-acetic acid; IAA, indole-3-acetic acid; IBA, indole-3-butyric acid; Ile, isoleucine; tiP, isopentenyladenine; Leu, leucine; LC-MS, liquid chromatography, mass spectrometry; NAA, 1-naphthaleneacetic acid; PAA, phenylacetic acid; Phe, phenylalanine; tDHZ, *trans*-dehydrozeatin; tDZR, *trans*-dehydrozeatin-riboside; tiP, *trans*-*N* ^6^-(Δ^2^-isopentenyl)adenine; tZ, *trans*-zeatin; tZ7G, *trans*-zeatin-7-glucoside; tZ9G, *trans*-zeatin-9-glucoside; tZOG, *trans*-zeatin-*O*-glucoside; tZR, *trans*-zeatin riboside; tZROG, *trans*-zeatin riboside *O*-glucoside; Trp, tryptophan; Val, valine.
