# Supplementary material for: Accumulation of and Response to Auxins in Roots and Nodules of the Actinorhizal Plant Datisca glomerata Compared to the Model Legume Medicago truncatula
Source: Front Plant Sci. 2019 Sep 24;10:1085. doi: 10.3389/fpls.2019.01085 (PMC6773980; doi:10.3389/fpls.2019.01085)

Supplementary Material

Accumulation of and response to auxins in roots and nodules of the actinorhizal plant *Datisca glomerata* compared to the model legume *Medicago truncatula*

Irina V. Demina, Pooja Jha Maity, Anurupa Nagchowdhury, Jason L. P. Ng, Eric van der Graaff, Kirill N. Demchenko, Thomas Roitsch, Ulrike Mathesius and Katharina Pawlowski

Supplementary Table S1.

Auxin and cytokinin levels in roots and nodules of *Medicago truncatula, Cicer arietinum* and *Datisca glomerata.* Values (pmol/g FW) in the tables represent average ± standard deviation. For the auxin profiles, significance of differences between concentrations in roots vs. nodules was determined using the Mann-Whitney U test. n.a. – Statistical test could not be conducted because one of the organs has insufficient data points, or no substance could be detected at all.

Medicago truncatula

	IAA	PAA	IAA-Ala	IAA-Asp	IAA-Leu/Ile	IAA-Phe	IAA-Trp	IAA-Val
**roots**	74.91 ± 48.63	119890.25 ± 44808.83	6.40 ± 2.95	67.77 ± 65.74	1.22 ± 0.50	0.92 ± 0.53	0.92 ± 0.26	0.00
**nodules**	126.53 ± 35.13	131257.95 ± 16619.45	91.22 ± 14.07	604.45 ± 81.93	1.14 ± 0.56	0.55 ± 0.40	2.41 ± 0.50	0.18 ± 0.07
**Mann-Whitney U test, p value**	0.250	0.393	0.036	0.057	1.000	0.413	0.057	n.a.

Cicer arietinum

	IAA	PAA	IAA-Ala	IAA-Asp	IAA-Leu/Ile	IAA-Phe	IAA-Trp	IAA-Val
**roots**	74.28 ± 15.37	55962.38 ± 18358.41	12.43 ± 4.29	1912.13 ± 640.21	0.00	0.32 ± 0.08	2.26 ± 1.34	0.00
**nodules**	294.16 ± 20.68	29068.71 ± 5575.24	18.19 ± 2.33	13149.48 ± 1903.95	0.21 ± 0.04	6.60 ± 1.57	28.98 ± 3.72	1.34 ± 0.43
**Mann-Whitney U test, p value**	0.016	0.016	0.064	0.016	n.a	n.a	0.016	n.a

Datisca glomerata

	IAA	PAA	IAA-Ala	IAA-Asp	IAA-Phe	IAA-Val
**roots**	10.51 ± 3.05	450.02 ± 161.66	4.99 ± 0.44	29.29 ± 22.12	3.20 ± 2.79	5.84 ± 0.83
**nodules**	8.55 ± 1.47	540.35 ± 372.58	9.78 ± 3.14	0.00	0.00	0.00
**Mann-Whitney U test, p value**	n.a.	0.904	0.036	0.016	n.a.	n.a.

Medicago truncatula

	tZ	cZ	iP	tDHZ	tZ7G	tZ9G	tZOG	tZROG	tDZR	tZR
**roots**	0.80 ± 0.41	0.16 ± 0.03	0.00	3.38	2.69	1.06	1.29	1.29 ± 0.24	0.00	0.07 ± 0.04
**nodules**	1.35	0.37 ± 0.02	0.00	0.00	8.12	0.69 ± 0.39	1.10 ± 0.23	1.10 ± 0.16	0.095 ± 0.04	0.80 ± 0.36

Datisca glomerata

	tZ	cZ	iP	tDHZ	tZOG	tZROG	tDZR	tZR
**roots**	1.43 ± 0.94	1.09 ± 0.48	3.12 ± 0.56	3.08 ± 0.49	3.37 ± 2.32	0.44 ± 0.09	0.13 ± 0.04	0.82 ± 0.42
**nodules**	3.47 ± 1.27	3.76 ± 1.21	2.25 ± 0.35	4.24 ± 0.76	95.70 ± 9.04	9.21 ± 2.08	0.14 ± 0.06	5.55 ± 3.87

Supplementary Figure S1. Growth of *Medicago truncatula* and *Datisca glomerata* in the presence of exogenous IAA or PAA. The photographs show the plants used to analyse the effects of NAA and PAA on root growth and -branching at the time of evaluation.

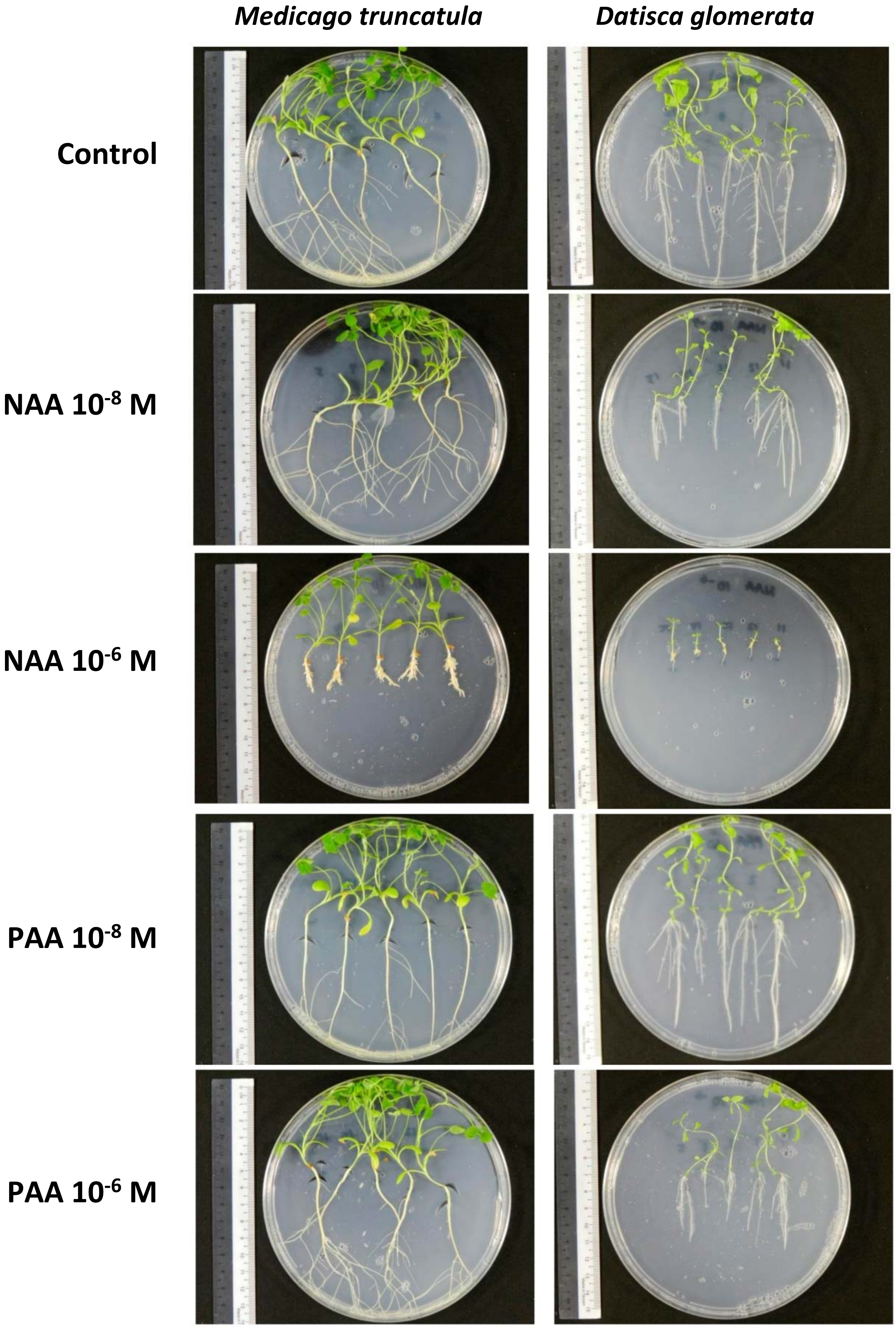

Supplementary Figure S2. Effect of NAA and PAA on root branching in *Medicago truncatula* and *Datisca glomerata*.

Panels **(A)** and **(B)** separate the number of emerged lateral roots per main root **(A)** from that of unemerged lateral roots per main root **(B)**.

Panel **(C)** shows lateral root density – emerged and unemerged lateral roots per cm main root; panel **(D)** shows emerged lateral roots per cm main root, while panel **(E)** shows unemerged lateral roots per cm main root.

Values represent means ± SEM. One-way ANOVA with Tukey's post hoc test was used to assess significant differences between treatment groups. Values labelled with the different letters are significantly different ($p \leq 0.05$). n = 9 to 12.

A

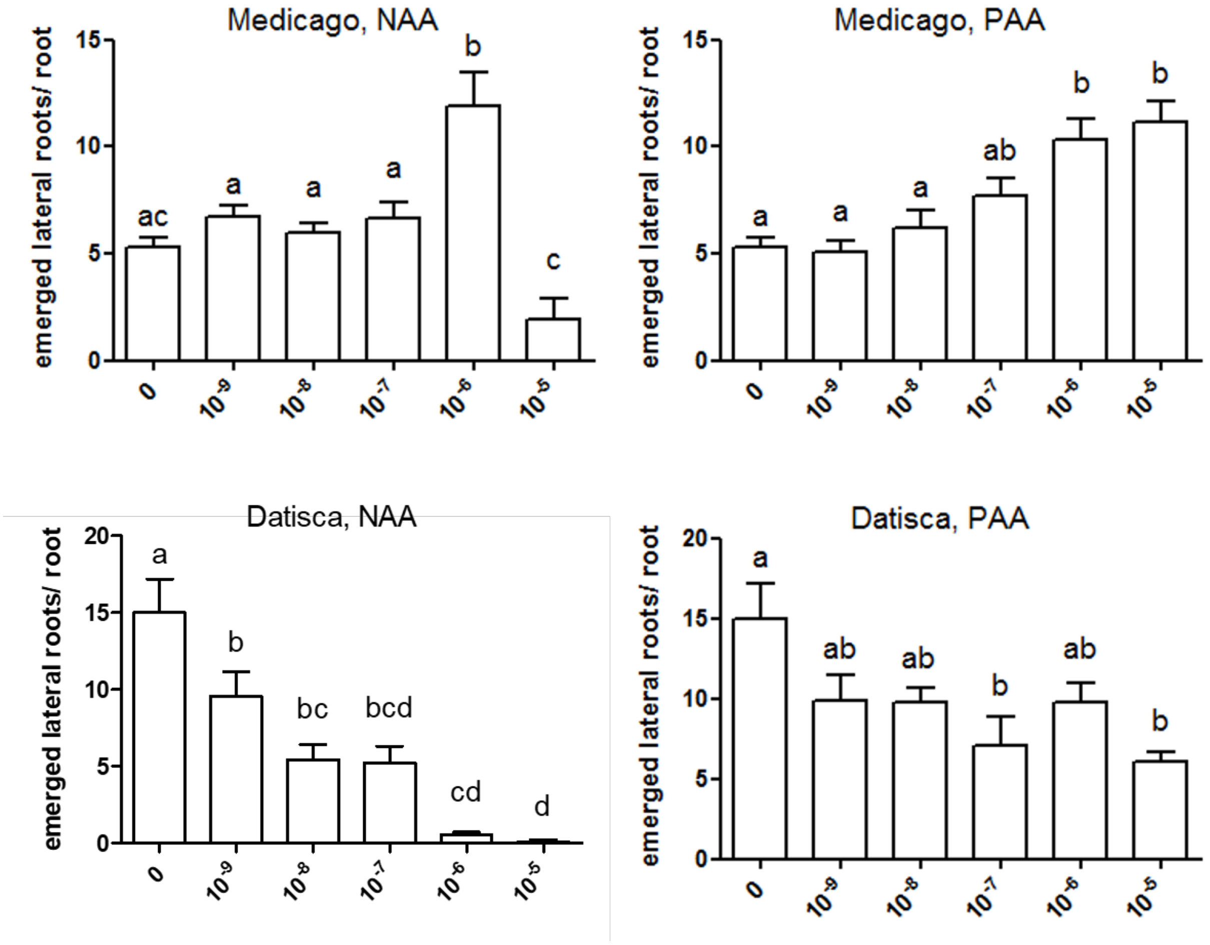

B

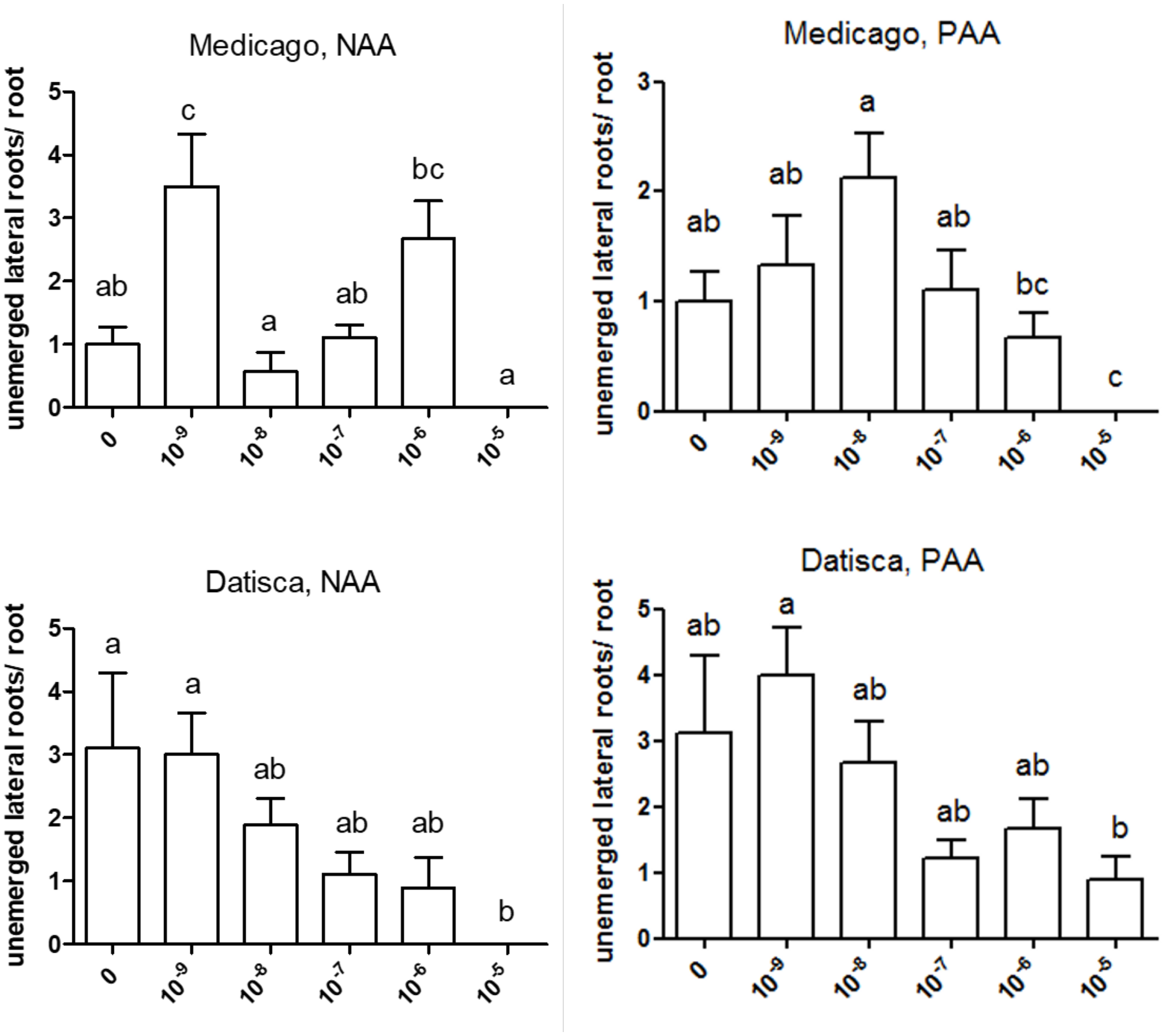

Medicago, NAA
unemerged lateral roots/ root
0
1
2
3
4
5
ab
c
a
ab
bc
a
0
10^-9
10^-8
10^-7
10^-6
10^-5
Medicago, PAA
unemerged lateral roots/ root
0
1
2
3
ab
ab
a
ab
bc
c
0
10^-9
10^-8
10^-7
10^-6
10^-5
Datisca, NAA
unemerged lateral roots/ root
0
1
2
3
4
5
a
a
ab
ab
ab
b
0
10^-9
10^-8
10^-7
10^-6
10^-5
Datisca, PAA
unemerged lateral roots/ root
0
1
2
3
4
5
ab
a
ab
ab
ab
b
0
10^-9
10^-8
10^-7
10^-6
10^-5

C

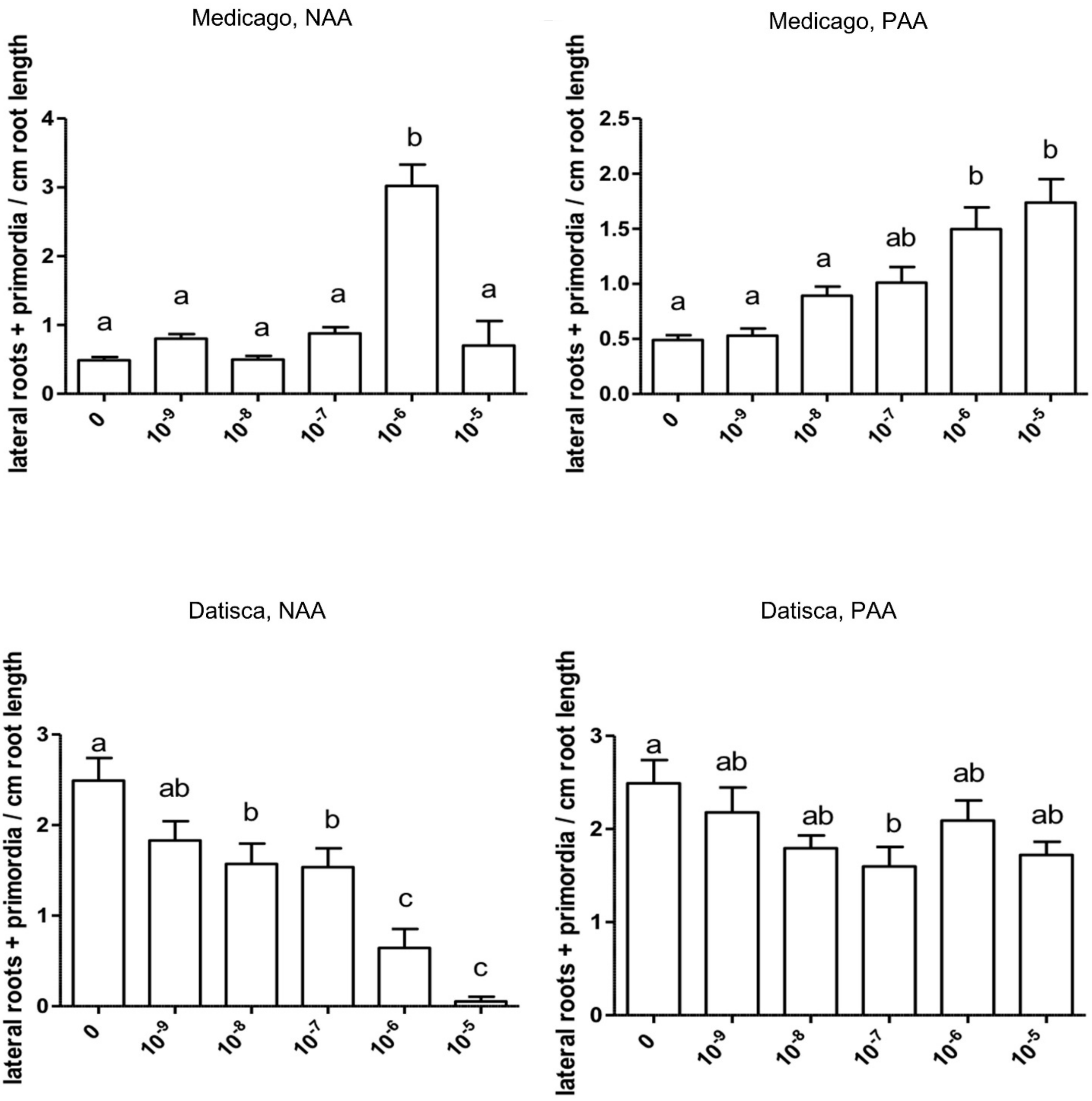
Medicago, NAA
Medicago, PAA
Datisca, NAA
Datisca, PAA
lateral roots + primordia / cm root length
0
10^{-9}
10^{-8}
10^{-7}
10^{-6}
10^{-5}
a
ab
b
c

D

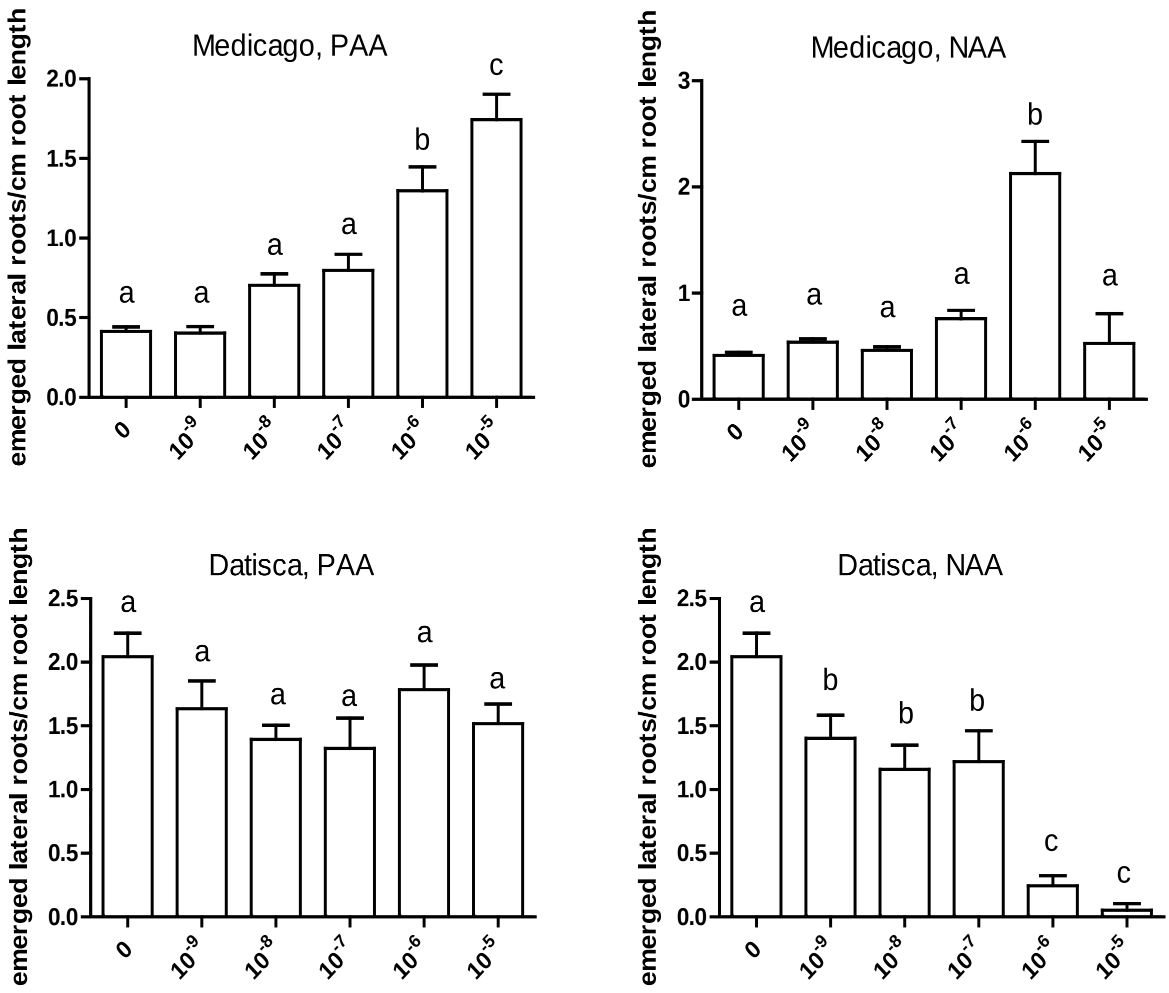
Medicago, PAA
Medicago, NAA
Datisca, PAA
Datisca, NAA
emerged lateral roots/cm root length
0
10-9
10-8
10-7
10-6
10-5

E

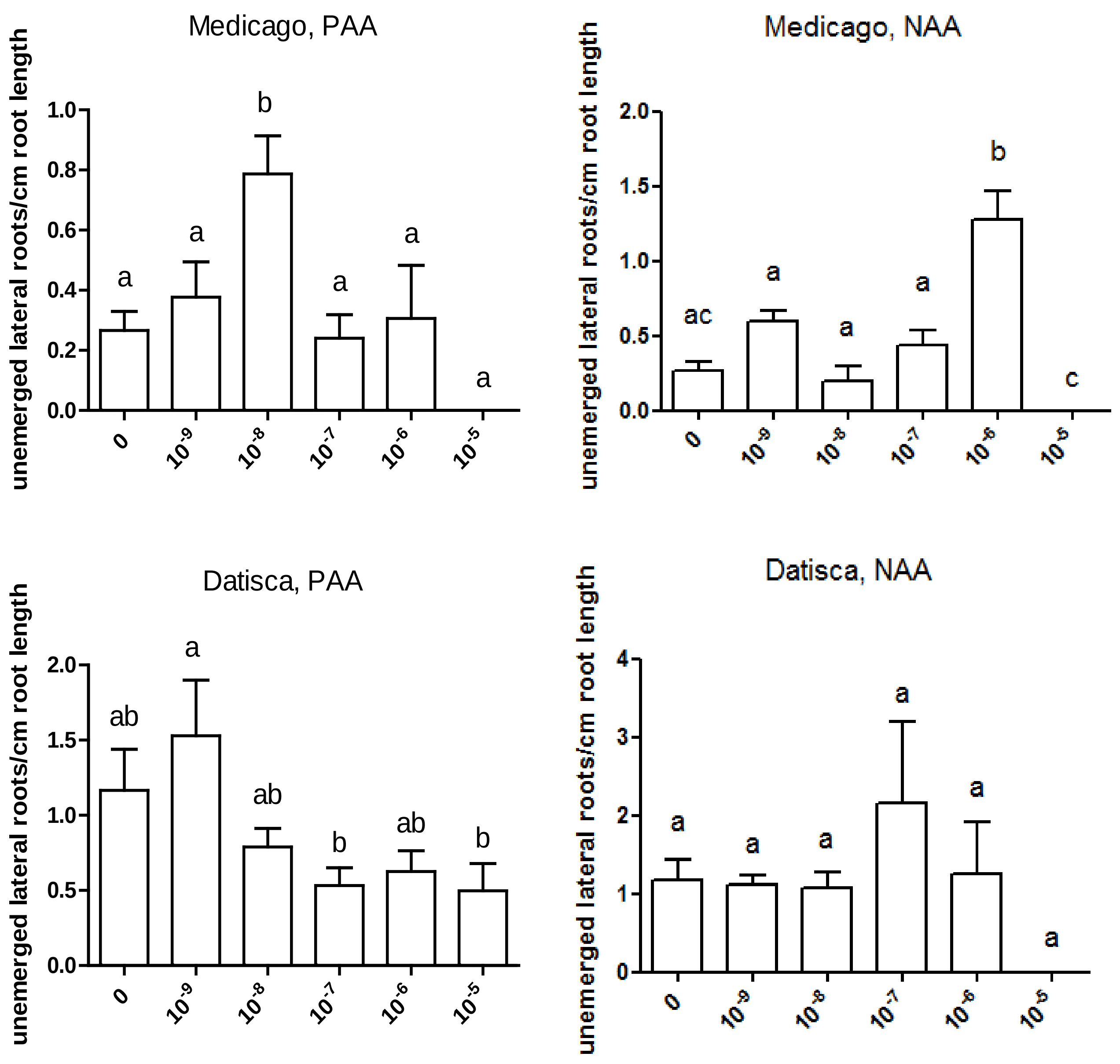
Medicago, PAA
unemerged lateral roots/cm root length
0.0
0.2
0.4
0.6
0.8
1.0
a
a
b
a
a
a
0
10-9
10-8
10-7
10-6
10-5
Medicago, NAA
unemerged lateral roots/cm root length
0.0
0.5
1.0
1.5
2.0
ac
a
a
a
b
c
0
10-9
10-8
10-7
10-6
10-5
Datisca, PAA
unemerged lateral roots/cm root length
0.0
0.5
1.0
1.5
2.0
ab
a
ab
b
ab
b
0
10-9
10-8
10-7
10-6
10-5
Datisca, NAA
unemerged lateral roots/cm root length
0
1
2
3
4
a
a
a
a
a
a
0
10-9
10-8
10-7
10-6
10-5

Supplementary Figure S3.

Adventitious roots and lateral root primordia of *Datisca glomerata*.

Arrows point at adventitious roots, distinguishable from lateral roots by their paucity of root hairs. **(A)** 4-week-old old plantlet grown on 10^{-5} M NAA, **(B)** 3-week-old plantlet grown on 10^{-5} M NAA. Adventitious roots clearly emerge not only from the hypocotyl, but also from the stem, and very sporadically. **(C)** Unemerged lateral root primordium. The root of a 4-week-old old *D. glomerata* plantlet grown on 10^{-6} M NAA was stained with Methylene blue to demonstrate how lateral root primordia were quantified.

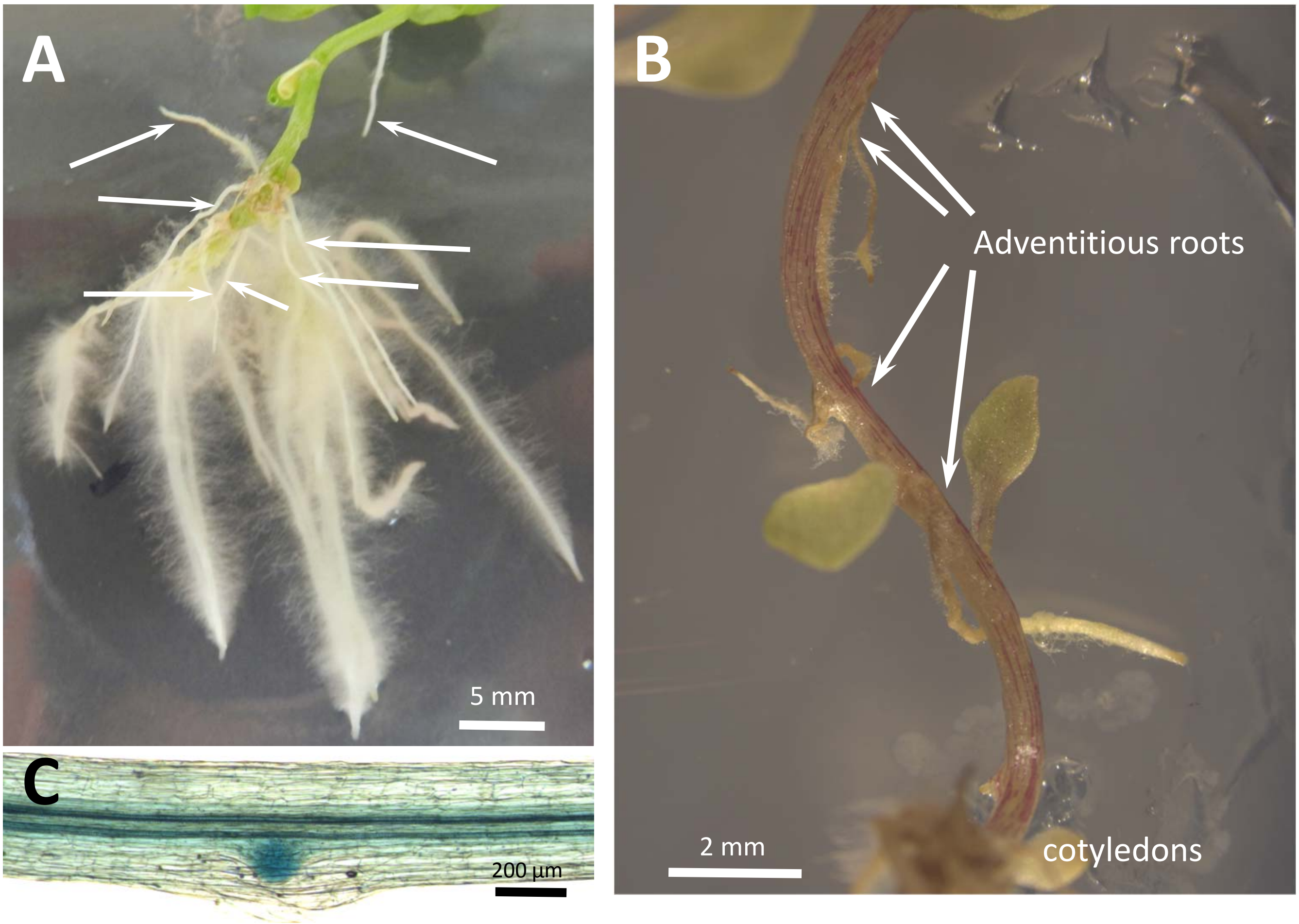

Supplement: Supplementary file 1 [file DataSheet_1.pdf]
